# Tuning the Photophysical Properties of Nickel and Zinc Complexes of N‑Confused Tetraphenylporphyrin via Trans–Cis Isomerization

**DOI:** 10.1021/acs.jpca.5c02035

**Published:** 2025-06-26

**Authors:** Eleftherios Papamichalis, Ioannis D. Petsalakis, Demeter Tzeli

**Affiliations:** † Laboratory of Physical Chemistry, Department of Chemistry, 68993 National and Kapodistrian University of Athens, Panepistimiopolis Zografou, Athens 157 84, Greece; ‡ Theoretical and Physical Chemistry Institute, National Hellenic Research Foundation, 48 Vassileos Constantinou Avenue, Athens 116 35, Greece

## Abstract

Porphyrins are detected in many biological systems and have significant roles in some important artificial systems, while the N-confused porphyrins present very interesting photophysical and chemical properties, which differ from those observed in porphyrins. In the present study, metal (M) complexes of tetraphenylporphyrin (TPP), N-confused TPP (NCTPP), and the ethenyl-pyrazine derivative of NCTPP (NCTPP-p), i.e., M-TPP, M-NCTPP, and M-NCTPP-p, where M = Zn^II^ and Ni^II^, were studied via density functional theory (DFT) and TD-DFT calculations. The photophysical properties of molecules and their absorption spectra are studied. It has been found that the M^II^ affects the relative stability of the M-NCTPP and M-NCTPP-p tautomers, resulting in different tautomers having the lowest energy structure, while for the M-NCTPP-p molecule, there are cis isomers, which are lower in energy than the corresponding trans isomers due to the van der Waals interactions. The global minima of the nickel complexes have the H atom of a reversed pyrrole attached to C (**a**), while the zinc complexes have the H atom outside of the porphyrin core attached to N (**b**). For M-NCTPP-p, the global minimums are **a.cis** (Ni^II^) and **b.trans** (Zn^II^). The absorption main peaks of M-NCTPP-p are red-shifted compared to M-NCTPP up to 80(135) nm for the Soret­(Q) bands. The different isomers present shifts of their main absorption peaks up to 50(180) nm. Additionally, the vertical de-excitation energies from selected excited states are also investigated. Overall, the selection of the metal and the peripheral group lead to different lowest values in the energy structure, affect its UV–vis absorption spectrum, and thus they tune the photophysical properties of the M-NCTPP complexes.

## Graphical abstract

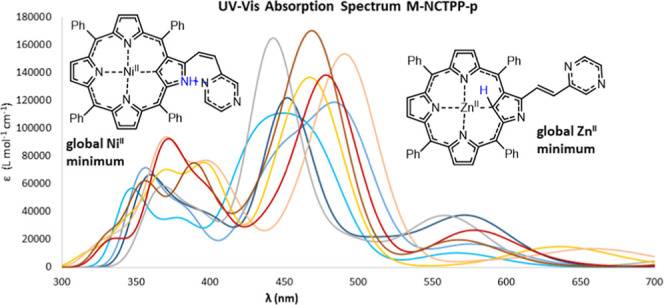

## 1 Introduction

Porphyrins are heterocycle organic compounds based on porphin. They are involved in important biological systems,^1–4^ while they present significant applications in chemistry, physics, medicine, and material science.^1–14^ Specifically, they have a primary or secondary role in the function and structure of relevant biological porphyrins, i.e., binding, storage, and transportation of oxygen, activation of molecular oxygen for oxidation in cytochrome P-450, absorption of solar energy as part of the chlorophyll molecule, transportation of energy, etc.^4–7^ Compounds of porphyrin are synthesized during the chlorophyll’s disintegration, a pigment substance found in greenery and in some bacteria.^1^ Furthermore, they have many application in material sciences, in transformation of solar energy, in optical and electric devices, etc.^8–13^ Additionally, they can serve as metal detectors due to their beaming in the presence of suitable molecule samples.^14^ Note that the metal complex of porphyrins is an extremely steady organic metallic molecule, where the metals are located in the center of the porphyrin bonded to the four nitrogen atoms of the pyrrole groups.

Nitrogen-confused porphyrin (NCP) is formed when one pyrrole is reversed. In 1994, Futura^15^ and Latos-Grazynski^16^ announced this reversion, while the NCP molecule has the same structure as the porphyrin. In this occasion, three nitrogen atoms and one carbon atom are in the core of the porphyrin. The hydrogen atoms can be either in the core of the porphyrin or at the outer peripheral of the reversed pyrrole ring. As a result, hydrogen atoms’ place induces new tautomers, which have different characteristics, such as photochemical quality and aromaticity.^15–29^ Like in plain porphyrins, a metal can be a compound in the core of the N-CP and bond with a carbon atom, while the peripheral nitrogen atom can act as a hydrogen-bonding donor or acceptor, which is crucial for the formation of multiporphyrin systems.^25–29^ It is interesting that NCPs have a remarkable ability to bind to a wide variety of cations in their free base and anions in their protonated forms.^21–26^ They present versatile coordination modes and can stabilize even some rare high oxidation states of metal ions.^21–29^ Finally, it should be noted that doubly NCPs have been synthesized, i.e., two pyrrole groups are inverted.^30,31^

The present paper is a continuation of our previous work on the photophysical properties of alkali metal complexes of NCP^18,19^ . The effect of the alkali metal cation on the geometry of the complexes and their bonding, as well as the effect of alkali metal and on their corresponding absorption spectra were studied. Here, nickel­(II) and zinc­(II) complexes of tetraphenylporphyrin (TPP) and of N-confused TPP (NCTPP), i.e., M-TPP and M-NCTPP, where M = Ni^II^ and Zn^II^, have been calculated, see Scheme 1. As mentioned above, the reverse pyrrole ring induces two tautomers **a** and b depending on the relative position of the H atom, M-NCTPP_a and M-NCTPP_b. Moreover, in addition to the tautomerism, the attachment on an ethenyl-pyrazine group (p) to an N-reversed pyrrole group induces a further trans–cis isomerism, i.e., M-NCTPP-p_a.trans, M-NCTPP-p_a.cis, M-NCTPP-p_b.trans, and M-NCTPP-p_b.cis; see Scheme 1. Hydrogen bonds are formed in the cases of the cis isomers that affect the relative stability of cis and trans isomers. It was observed, in the case of the ethynyl-pyridine derivative, that the cis isomer of the Ni^II^ complex of NCP is stable.^20^ Note that pyridine is more basic than pyrazine, and as a result it can form a stronger H bond with the main ring than pyridine. In this study, the ethenyl-pyrazine group is chosen so as to check if the pyrazine that forms weaker H bonds than pyridine has this effect also for both Ni^II^ and Zn^II^ complexes. Note that TPP has no peripheral N atom that can be protonated and affects the trans–cis isomerization. As a result, the trans isomer will be the most stable structure in the case of the TPP.

**Scheme 1:**
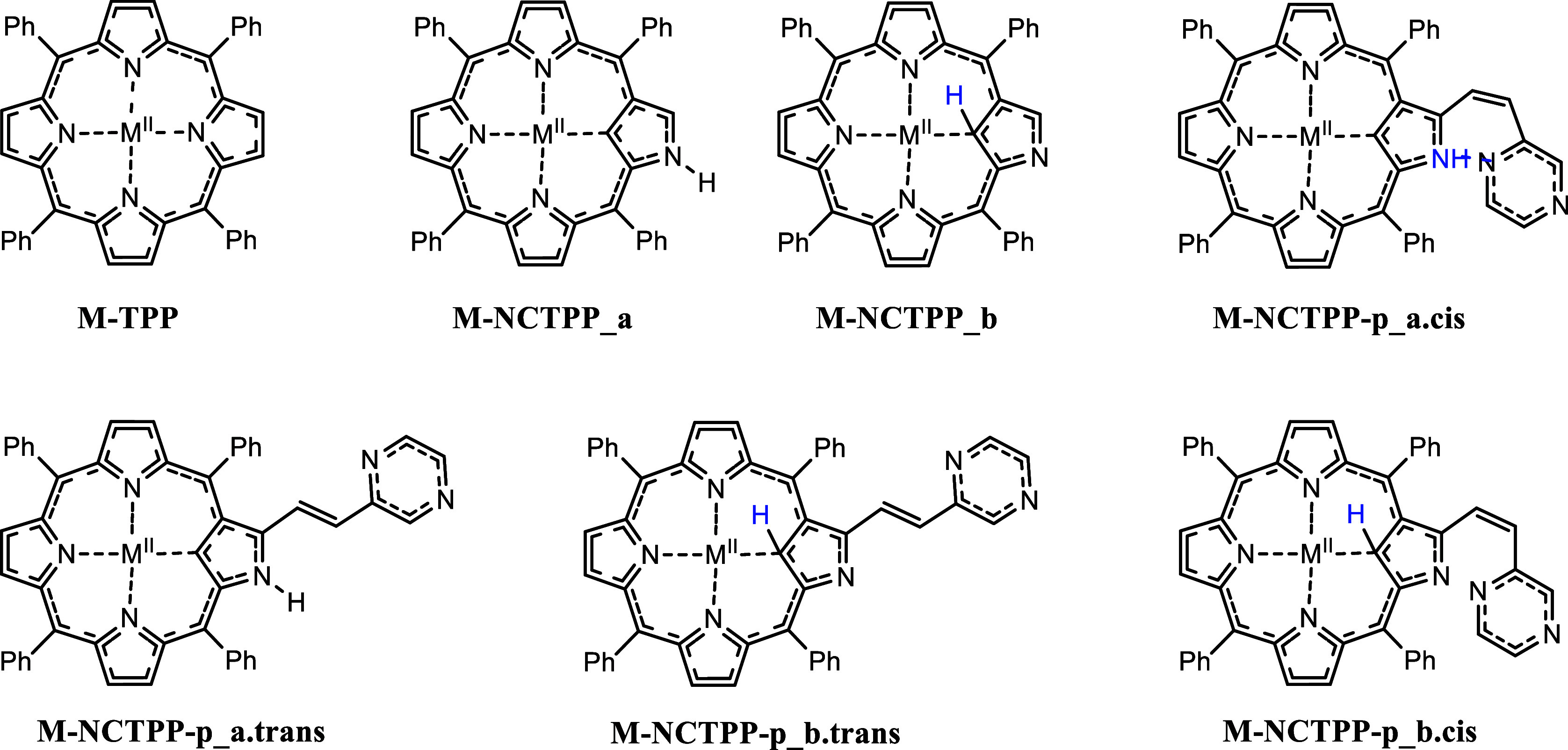
Metal Complexes of TPP, M-TPP, NCTPP, M-NCTPP, and Ethenyl-Pyrazine Derivative M-NCTPP-p

The main aim of the present work is the study of the photophysical properties of the studied complexes. This work aims to explain how the metal cation affects the relative stability of the M-TPP complexes, the M-NCTPP tautomers, and the M-NCTPP-p trans and cis isomers and tautomers. Furthermore, how the M cation affects their absorption spectrum results in a tunable complex regarding its photophysical properties and consequently in different applications such as optical memories, switches, and molecular logic gates at the molecular level. Finally, the de-excitation energies from selected low-lying excited states are also investigated.

## 2 Computational Details

The TPP (P), the NCTPP (NCP), and ethenyl-pyrazine derivative (NCP-p) and their corresponding metal (M) complexes, i.e., M-P, M-NCP, and M-NCP-p, where M = Ni­(II) and Zn­(II) have been calculated via density functional theory (DFT) and TD-DFT methodologies. Various isomers, i.e., tautomers, trans, and cis isomers, have been studied, and their photophysical properties were analyzed, while their absorption spectra were obtained. All calculations were carried out in dimethylformamide (DMF) solution employing the polarizable continuum model.^32,33^

At first, conformational analyses were carried out, where the geometry of the molecules were fully energetically optimized using the PBE0 ^34,35^ functional and the 6-31G­(d,p)^36^ basis set to locate the minimum structure for each of the 14 structures of Figure 2. Then their frequencies were calculated to ensure that the calculated structures are true minima. Additionally, Mulliken analysis, charge Model 5 (CM5) which is an extension of Hirshfeld population analysis,^37^ and natural population analysis (NPA)^38^ were used to calculate transition metal charges. The ground state of the molecules is a singlet state. The stability of the wave function was checked. Geometry calculations for a triplet state for both metals confirm that the singlet states are the lowest ones.

To study the electronic structure and the photophysical properties of the molecules, the UV–vis absorption spectra of the studied structures were calculated including the 50 lowest in energy excited singlet-spin electronic states in the DMF solvent at TD-PBE0/6–31G­(d,p). The UV–vis peak half-width at half-height is 0.2 eV. Furthermore, the de-excitation energies for six significant excited states for nickel-complexes and eight for zinc-complexes were calculated.

Furthermore, the linear response correction (cLR) approach has been used for the calculation of the main absorption peaks and their de-excitation energy.^39,40^ cLR corrects for the density-dependent relaxation of solvent polarization. It was found that the inclusion of this approach affects only the de-excitation energies, while it does not affect the absorption peaks of the present calculated complexes.

It should be noted that the methodology used, PBE0/6-31G­(d,p), has been considered as appropriate for the calculation of the present molecules. The PBE0 functional has been extensively tested for its ability to predict excited state properties, including vertical excitation energies and excited state geometries.^41,42^ Specifically, PBE0’s performance compared to 17 other functionals for predicting vertical excitation energies of singlet excited states has shown that PBE0 is among the most effective functionals regarding the average deviation, i.e., the mean absolute error is about 0.22 eV.^41^ Additionally, in the cases of transition metal complexes, PBE0 performs well for various metal porphyrin complexes.^43^ Furthermore, the methodology used, TD-PBE0/6-31G­(d,p), has been applied successfully for the study of the photophysical properties and the calculation of UV–vis absorption and emission spectra of transition metal complexes.^43–49^

However, for reasons of comparison, additional geometry optimization has been carried out at PBE0/6-311G­(d,p)^36^ methodology for the M-NCTPP and it was shown that both 6-31G­(d,p) and 6-311G­(d,p) basis sets resulted in the same geometries. Additionally, geometry optimizations were carried out for selected M-NCTPP-p complexes at the PBE0-D3 ^50^/6-31G­(d,p); it was found that the inclusion of the D3 dispersion correction^50^ resulted in similar geometries. The geometries are given in the Supporting Information. Finally, the absorption spectra of selected M-NCTPP-p complexes were calculated via the CAM-B3LYP^51^/6-31G­(d,p) methodology for reasons of comparison.

All DFT calculations were carried out with the Gaussian 16 software package.^52^

## 3 Results and Discussion

The present study aims to study the effect of the metal cation on the relative stability and the photophysical properties of the nickel and zinc complexes of N-confused tetraphenyl porphyrin derivatives. In what follows, geometry differences of the metal complexes are highlighted, followed by the energetics, UV–vis absorption spectra, de-excitation energies, and frontier molecular orbitals (MOs).

### 3.1 Geometry

The calculated structures of the M-TPP, M-NCTPP, and M-NCTPP-p complexes, including the trans and cis isomers and tautomers, are depicted in Figures 1 and S1 of the Supporting Information. In all M-NCTPP and M-NCTPP-p complexes, there are two tautomers, i.e., the **a** tautomer which has the H of the reversed porphyrin bonded at the N atom, while the **b** tautomer has this H inside the porphyrin core and as a result, the **b** tautomers have a more deformed core than the **a** tautomers.

**Figure 1:**
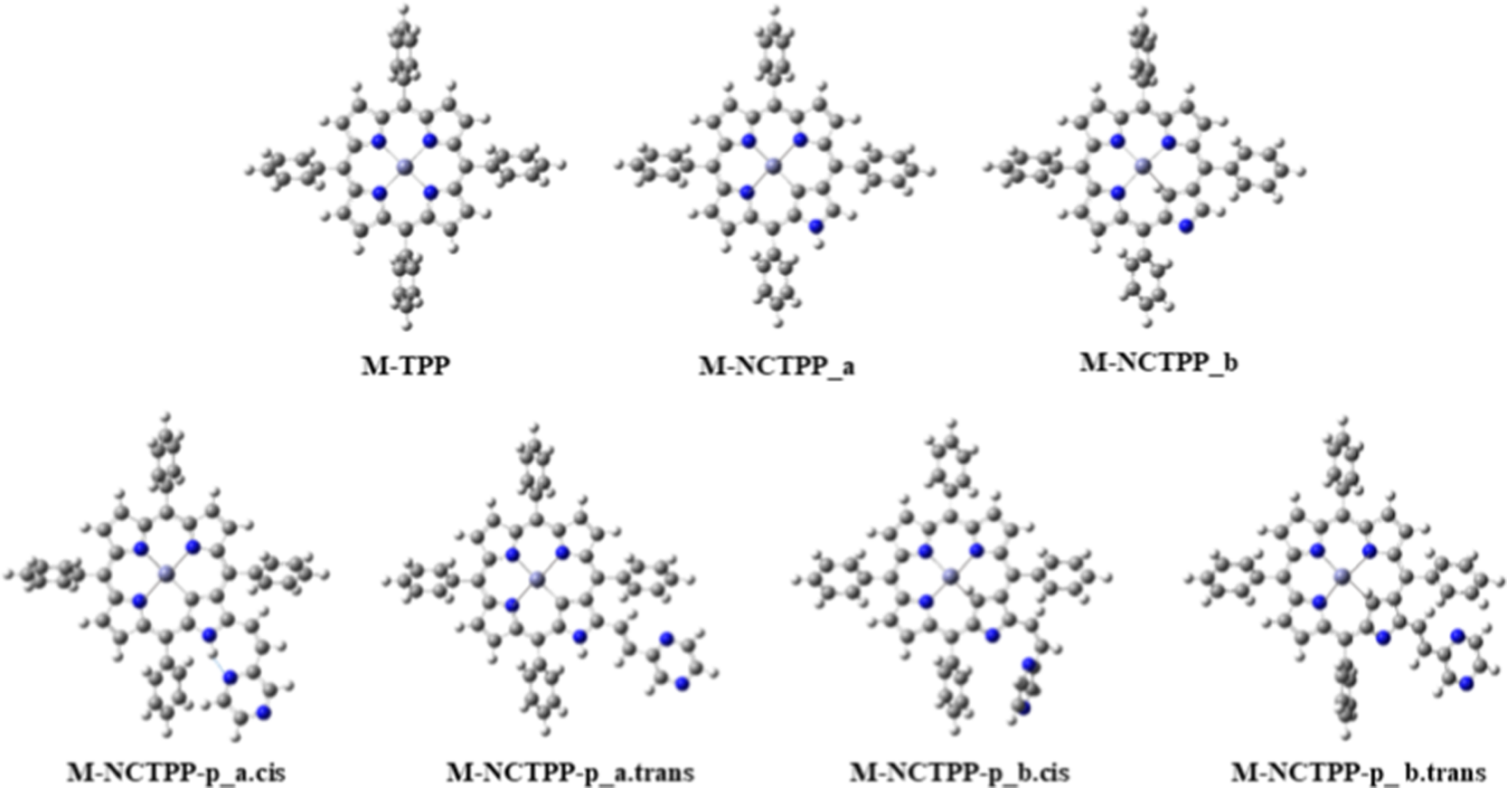
Metal complexes of TPP, M-P, NCTPP, M-NCP (M-NCP_a and M-NCP_b tautomers) and ethenyl-pyrazine derivative M-NCP-p (M-NCP-p_a.trans, M-NCP-p_a.cis, M-NCP-p_b.trans and M-NCP-p_b.cis), where M = Ni^II^ and Zn^II^.

The M-N and M–C bond distances of the M-NCTPP and M-NCTPP-p are given in SI and are depicted in Figure 2i. For all Ni^II^ and Zn^II^ complexes, **b** tautomers present M–N bond distances shorter than those of the corresponding **a** tautomers. On the contrary, **a** tautomers present the shortest M–C bond distances than the corresponding **b** tautomers. Both trends are due to the existence of the H atom bonded to the inner C atom at the **b** tautomers, and thus, the M is shifted closer to the N atoms and farther than the C atom of the reversed pyrrole ring. Specifically, the C–Ni bond distance is 1.927 Å in the Ni-NCTPP_a, while it is elongated by 0.14 Å in the Ni-NCTPP_b, i.e., it is 2.069 Å. In the Zn^II^ complexes, this elongation is increased. The corresponding values are 1.986 and 2.317 Å, and the elongation is significantly larger, i.e., 0.33 Å. This occurs due to the larger size of the Zn^II^ compared to the Ni^II^, i.e., the ionic radius of Zn^II^ is larger than the ionic radius of Ni^II^,^53^ and due to the fact that all d orbitals of the Zn^II^ are fully occupied, i.e., the electron charges on 3d orbitals in 9.9 e^–^, see Table S5 of the Supporting Information. Thus, Ni^II^ has a higher effective nuclear charge on the remaining electrons than Zn^II^, so it pulls them in more tightly, while the fully filled 3d orbitals in Zn^II^ add more electron–electron repulsion, slightly increasing the radius. Finally, regarding the N–M distances, the differences of the N–Ni distances between **a** and **b** tautomers are about 0.03 Å and significantly smaller in Zn^II^ complexes, see Table S2 of the Supporting Information and Figure 2.

**Figure 2:**
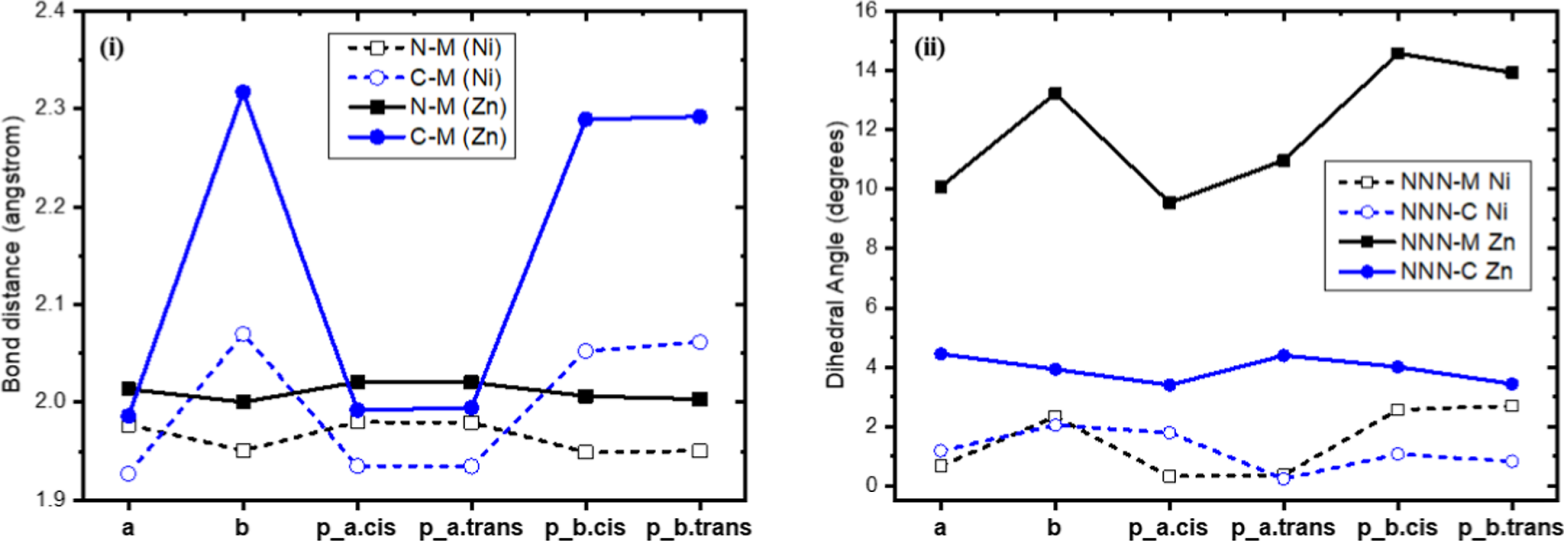
(i) Bond distance between the metal and the nearest nitrogen or carbon atom of the porphyrin core. (ii) NNNM and NNNC dihedral angles of porphyrin’s core of the M-NCTPP and M-NCTPP-p.

Nickel is almost located in the NNN plane, i.e., the NNNNi dihedral angle ranges from 0.3 to 2.7°, while zinc is above the NNN plane, i.e., the NNNZn dihedral angle ranges from 9.5° to 14.6 Å. At the **b** tautomers, the M declines from the NNN plane more than at the **a** tautomers, see Figure 2ii.

Moreover, the effect of inclusion of the empirical D3 dispersion correction in the calculation of the geometry was investigated. It was found that its inclusion resulted in the same general geometry. The inclusion of the dispersion correction resulted in shorter M–N and M–C bond distances, the bond length reduction ranges from 0.002 Å to 0.08 Å, see Table S4 of the Supporting Information. The largest reduction was observed only for the M–C bond distances in the cases of the **b** tautomers, where the H is bonded to the C core atom. However, both PBE0 and PBE0-D3 present the same CNNN and MNNN dihedral angles and MCH angles, showing that D3 does not change the M-core geometry.

The Mulliken, CM5, and NPA charges have been calculated. All population analyses show that both Ni^II^ and Zn^II^ have a positive charge and Zn^II^ is the most positive one. The NPA charge on Ni is about 0.9 e^–^ and the charge on Zn is about 1.4 e^–^, see Tables S5 of the Supporting Information. The electron density on M is Ni: 4s^0.4^3d^8.4^4p^0.3^ and Zn: 4s^0.4^3d^9.9^4p^0.3^ showing that the bond is formed from Ni^II^(4s^0^3d^8^) and Zn^II^(4s^0^3d^10^). Due to the bond formation between the M^II^ and the porphyrin core, electron charge is transferred from the N atoms to the M^II^, i.e., about 0.4 e^–^ is transferred to the 4s, 0.3 e^–^ is transferred to the 4p, while in the case of the nickel, 0.4 e^–^ is transferred to its half-occupied d electron. This is the reason that Ni has a less positive charge than Zn.

### 3.2 Energetics

#### 3.2.1 M-TPP and M-NCTPP

The relative energy differences of the calculated minima structures are depicted in Figure 3. The effect of the metal on the relative stability of the calculated isomers is very interesting. For both metals, the plain M-TPP is more stable than the lowest in energy M-NCTPP by 20.15 kcal/mol (Ni^II^) and 26.61 kcal/mol (Zn^II^), see Figure 3i. However, different tautomer of the M-NCTPP complexes is the lowest one for each metal, i.e., the **a** tautomer for the Ni^II^, which has the H of the reversed porphyrin bonded at the N atom, while the **b** tautomer for the Zn^II^, which has this H inside the porphyrin core, see Scheme 1. Note that in the case of the free NCP, i.e., without a metal, Furuta and co-workers^54^ experimentally separated its two tautomers. Specifically, the two tautomers, NCP-3H and NCP-2H species, were separated using solvents of different polarities, and they measured distinct absorption spectra. The NCP-3H corresponds to the **b** tautomer and NCP-2H to the **a** one. Specifically, NCP-3H could be isolated in dichloromethane (DCM), i.e., a poorly polar solvent, while NCP-2H was isolated in *N*-*N*-DMF, a highly polar solvent. A color change also was observed in the NCP solutions, red in DCM and green in DMF. For the NCTPP, the NCTPP-3H is more stable than **b** by ∼3 kcal/mol depending on the solvent.^18^

**Figure 3:**
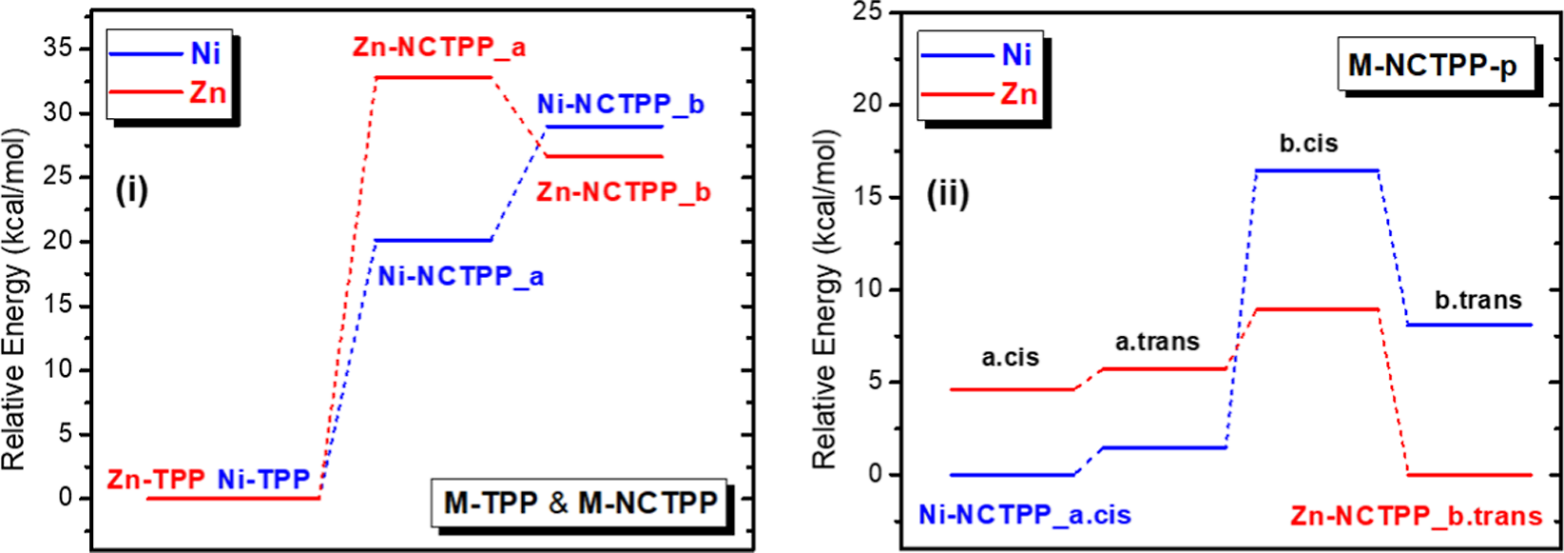
Relative energy differences of the different isomers of: (i) M-TPP & M-NCTPP and (ii) M-NCTPP-p complexes.

Here, it was found that for Ni^II^, the **a** tautomer is more stable than the **b** one by 8.15 kcal/mol, while for Zn^II^, the ordering is reversed, i.e., the **b** tautomer is more stable than the **a** one by 6.21 kcal/mol, see Table S1 of the Supporting Information and Figure 3i. This difference between the two metals results from their electronic structure, which strongly affects their bonding and the minimum structures of its complexes.^55^ Zn^II^ has a d^10^ electronic configuration, while Ni^II^ has a d^8^ electronic configuration having unpaired d electrons, see the discussion above. Note that the free NCP has the corresponding **b** tautomer as the most stable one (NCP-3H), which is regarded as strongly aromatic as a regular porphyrin. Thus, the full paired Zn^II^ does not change the relative ordering of the **a** and **b** tautomers of the free NCTPP. On the contrary, the deficiency of the d electrons of Ni^II^ attracts charge density from the NCTPP, and this the reason why the nickel charge is about 0.9 e^–^ in the complex. Thus, Ni^II^ forms stronger bonds with the N core atoms; however the H atom in the core impedes it, and this is the reason why it is favoring the H atom to be bonded in the reversed N atom, resulting in a more stable **a** structure than the **b** one.

#### 3.2.2 M-NCTPP-p Complexes

The ordering of the **b** and **a** tautomers of the M-NCTPP is retained at the M-NCTPP-p complexes, cf. Figure 3i,ii. Thus, a **b** tautomer, i.e., **b.trans**, is the lowest in energy for the Zn^II^ complex and the **a** tautomer, i.e., **a.cis**, is the lowest one for the Ni^II^ complex. Note that the ethenyl-pyrazine group induces a cis–trans isomerization and the relative ordering starting from the most stable one is **b.trans** > **a.cis** > **a.trans** > **b.cis** for the Zn^II^ and is **a.cis** > **a.trans** > **b.trans** > **b.cis** for the Ni^II^ complex. Only, in the case of the **a.cis**, a hydrogen bond H···N is formed, resulting in the formation of a 7-member ring and a further stabilization of this isomer. As a result, the **a.cis** isomers are more stable than **a.trans** for both metals by about ∼1.3 kcal/mol, while the energy ordering of trans/cis isomers of **b** tautomers follow the opposite ordering, i.e., **b.trans** are more stable than **b.cis** for both metals by about 8.5 kcal/mol, see Table S1 of the Supporting Information and Figure 3.

### 3.3 Absorption UV–Vis Spectra

The electronic spectra of porphyrins present two main absorption regions, a rather weak band, named a Q-band, in the range of 550–700 nm and a strong absorption band, named a Soret or B band in the range of 250–500 nm.^56,57^ According to the four-orbital model theory, the four orbitals are the π-bonding and π* antibonding orbitals of a porphyrin. In the plain porphyrins, the two highest occupied orbitals (HOMO) have a symmetry of a_1u_ and a_2u_, and the two lowest unoccupied orbitals (LUMO) have a symmetry of e_g_. The two main forms of absorption are the transition coupling between HOMOs and LUMOs (π → π*). At the Q-band, the transition dipoles cancel each other out, resulting in a weak absorption band. On the contrary, the Soret transition is a linear combination of two transitions, reinforcing the transition dipole resulting in strong absorption.^56,57^ Generally, plain porphyrins have many applications, and the diversity of their functions is influenced by the variety of metals that bind within the porphyrin ring system.

The absorption spectra of the M-TPP, M-NCTPP, and M-NCTPP-p tautomers and isomers are plotted in Figures 4 and 5. It is found that the plain TPPs have absorption spectra with one strong peak, while NCTPPs have two main peaks. Adding the ethenyl-pyrazine group in NCTPP, additional peaks are added to the UV–vis absorption spectra. Energy differences Δ*Ε*, wavelengths λ, and *f*-values of the main absorption peaks of the Q and Soret bands are given in Table 1 with available experimental data.^58–62^ There is a good agreement between the available experimental data, i.e., the Δ*Ε* values of the calculated UV–vis absorption peaks are blue-shifted, and the shifts range from 0.08 to 0.2 eV. Note that shifts up to 0.2 eV are considered as reasonable, i.e., there is a good agreement between experimental and TDDFT calculations.^63^ It should be noted that the cLR correction does not affect the calculation of the absorption peaks.

**Figure 4:**
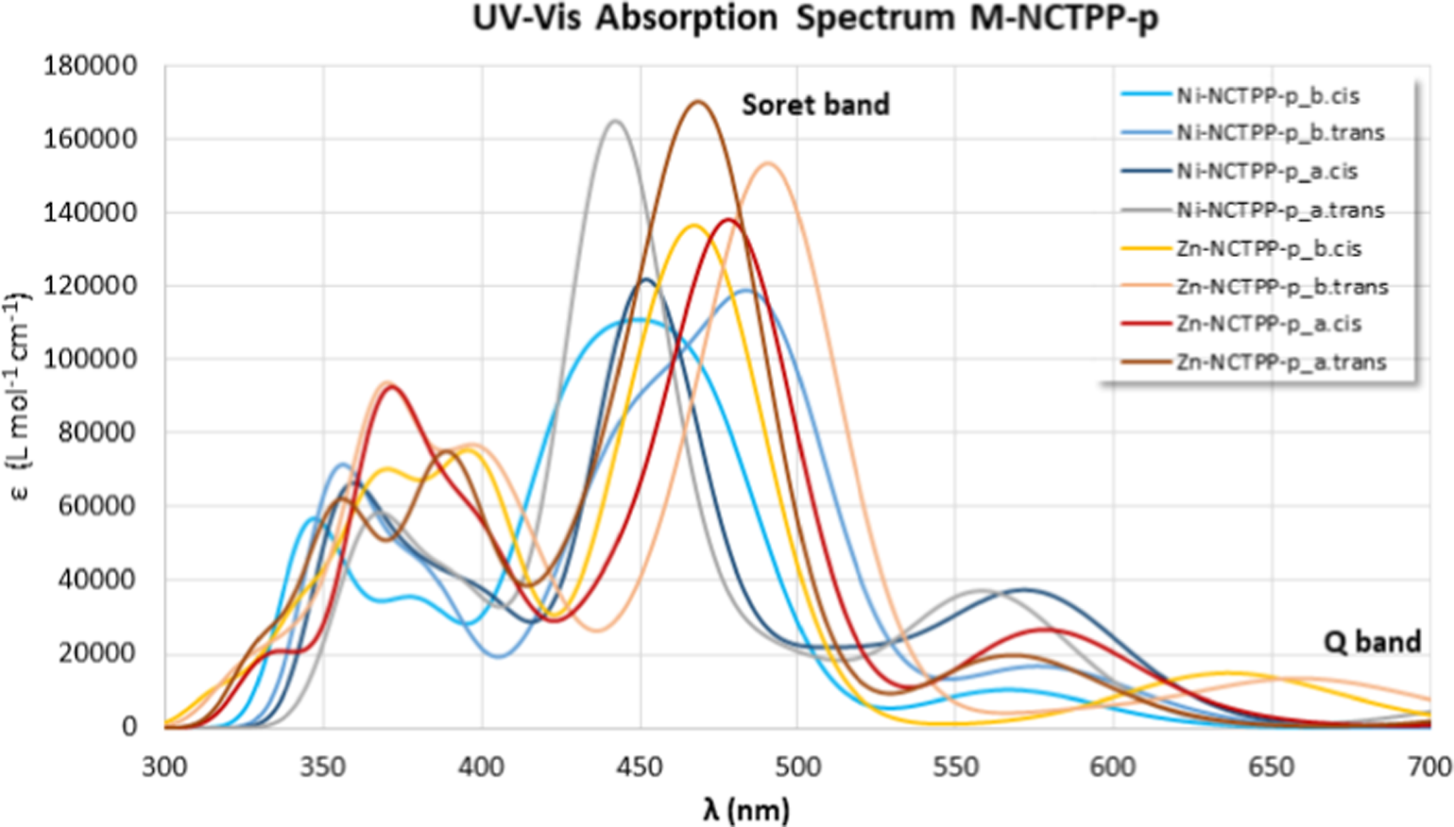
Absorption Spectra of the M-NCTPP and M-NCTPP-p tautomers and isomers at the PBE0/6-31G­(d,p) level of theory in the DMF solvent.

**Figure 5:**
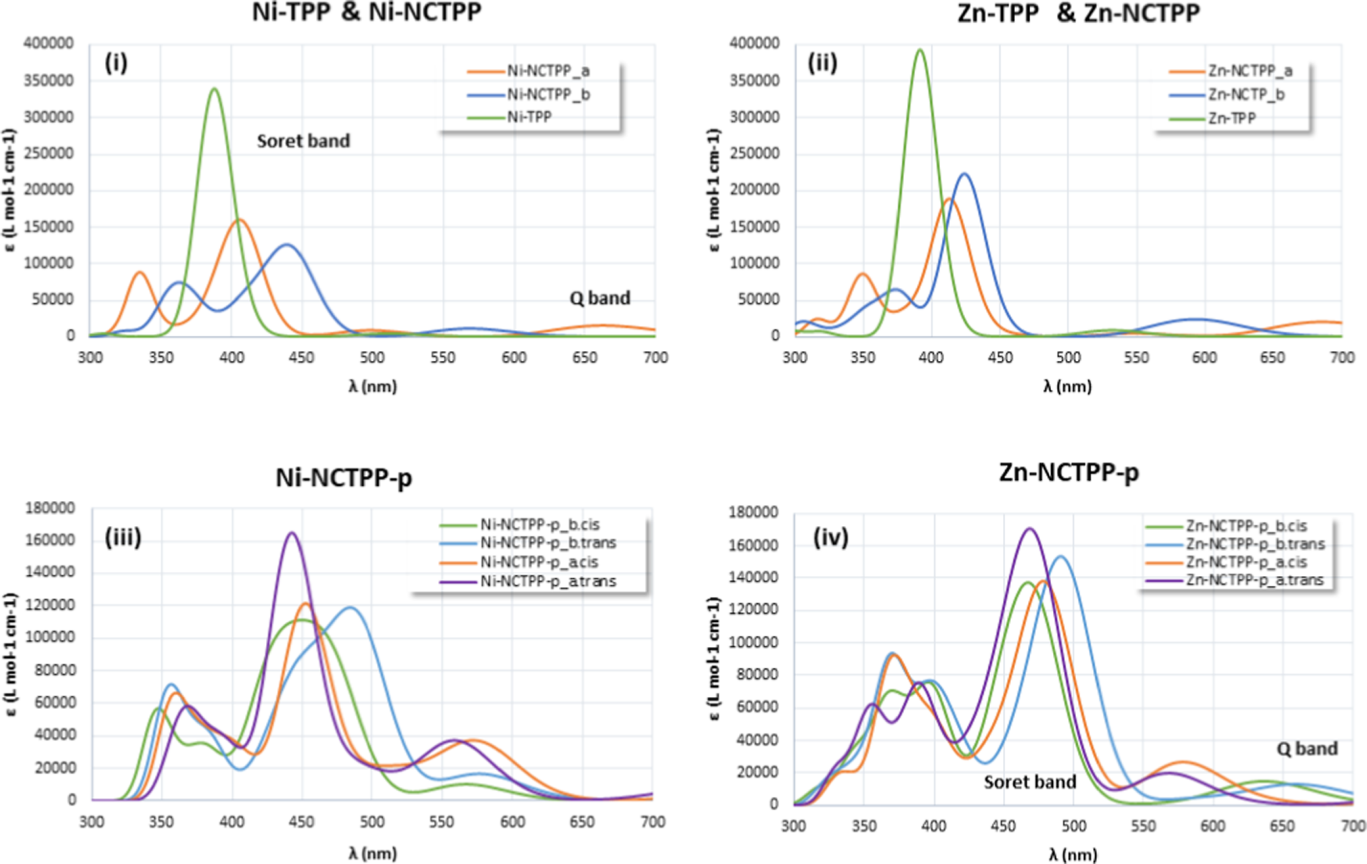
Absorption Spectra of the M-TPP, M-NCTPP, and M-NCTPP-p tautomers and isomers at the PBE0/6-31G­(d,p) level of theory in the DMF solvent.

**Table 1:** Energy Differences Δ*Ε* (eV), Wavelengths λ (nm), *f*-Values of the Main Vis–UV Absorption Peaks of the Q and Soret Bands of the M-TPP, M-NCTPP, and M-NCTPP-P Tautomers and Isomers at the PBE0/6-31G­(d,p) Level of Theory^f^

	λ	Δ*Ε*	*f*	λ	Δ*Ε*	*f*	λ	Δ*Ε*	*f*	λ	Δ*Ε*	*f*
	Ni-TPP						Zn-TPP					
	511.0	2.426	0.019				532.0	2.331	0.039			
	388.1	3.195	1.508				391.4	3.168	1.753			
Q^a^	527^b^	2.35					515 550^c^	2.25 2.41				
S^a^	415^b^	2.99					419^c^423^d^	2.93 2.96				
	Ni-NCTPP_a			Ni-NCTPP_b			Zn-NCTPP_a			Zn-NCTPP_b		
	658.5	1.883	0.093	572.5	2.166	0.058	685.1	1.810	0.187	603.0	2.056	0.153
	408.9	3.032	1.179	444.6	2.789	0.585	414.1	2.994	1.445	425.2	2.916	1.151
	391.6	3.166	0.482	441.3	2.810	0.364	350.8	3.535	0.630	421.6	2.941	0.858
	334.8	3.704	0.764	406.2	3.052	0.173	342.1	3.624	0.124			
				362.4	3.421	0.342				376.1	3.297	0.516
Q^a^	550–735^e^	1.69–2.25^e^		591^e^	2.10							
S^a^	426^e^	2.91^e^		467^e^	2.65							
	Ni-NCTPP_a.cis			Ni-NCTPP_b.cis			Zn-NCTPP_a.cis			Zn-NCTPP_b.cis		
	793.4	1.563	0.092	565.6	2.192	0.037	820.0	1.512	0.114	637.2	1.946	0.129
	575.7	2.154	0.307	474.8	2.611	0.535	577.6	2.147	0.233	473.4	2.619	0.977
	454.3	2.729	0.316	455.3	2.723	0.388	480.8	2.579	1.031	449.4	2.759	0.504
	451.3	2.747	0.765	438.8	2.826	0.386	447.7	2.769	0.239	399.5	3.103	0.592
	399.5	3.103	0.146	415.4	2.985	0.172	400.5	3.096	0.384			
	356.7	3.476	0.466	384.5	3.225	0.192	368.4	3.365	0.554	366.8	3.380	0.453
				344.6	3.598	0.366				347.3	3.570	0.141
	Ni-NCTPP_a.trans			Ni-NCTPP_b.trans			Zn-NCTPP_a.trans			Zn-NCTPP_b.trans		
	756.4	1.639	0.088	586.4	2.114	0.065	789.4	1.571	0.115	660.9	1.876	0.116
	559.9	2.214	0.322	491.2	2.524	0.878	567.3	2.186	0.171	493.1	2.514	1.282
	489.1	2.535	0.158	464.7	2.668	0.346	473.0	2.621	1.273	461.7	2.685	0.317
	444.0	2.792	1.114	446.8	2.775	0.383	443.9	2.793	0.375	417.7	2.968	0.252
	393.3	3.152	0.269	384.2	3.227	0.284	389.8	3.181	0.624	397.6	3.118	0.540
	369.4	3.356	0.192	352.8	3.514	0.475	353.9	3.503	0.327	370.9	3.343	0.654

a Experimental main peaks at the Q and Soret bands.

b References 56 and 57 ; in the CH_2_Cl_2_ solvent.

c References 59 and 60 .

d Reference 61 in the toluene solvent.

e Reference 62.

f Experimental values in italics.

The main Q peak of the Ni-TPP and Zn-TPP was calculated at 511 and 532 nm, in the DMF solvent, respectively. These values are in very good agreement with the experimental values of 527 nm in the CH_2_Cl_2_ solvent for Ni-TPP^56,57^ and 515 and 550 nm for Zn-TPP.^59^ For the corresponding confused porphyrins, the **a** tautomers of both metal complexes present a red shift of about 150 nm, while the **b** tautomers present red shifts of about 65 nm on the average with respect to the M-TPP porphyrins, see Table 1. For the M-NCTPP-p complexes, an additional red shift that ranges from 98 to 135 nm is observed at the **a** tautomers with respect to the **a** tautomers of the M-NCTPP. Note that the **a.cis** isomers present the larger red shifts than the **a.trans** ones. Additionally, the **a** tautomers of M-NCTPP-p present Q absorption peaks in the area of 560–578 nm with oscillator strengths of about 0.25. Finally, the **b** tautomers of M-NCTPP-p present a small red shift with respect to the M-NCTTP that range from −7 to 58 nm. The absorption spectrum of selected structures was calculated via CAM-B3LYP/6-31G­(d,p), see Table S6 and the Supporting Information. Both PBE0 and CAM-B3LYP predicted spectra with a similar shape; however the oscillator strengths of the CAM-B3LYP are larger than those of the PBE0 for the Soret bands, while for the Q bands, they are similar. The CAM-B3LYP calculates the Soret band blue-shifted by about 40 nm with respect to the corresponding Soret band calculated via the PBE0. This blue shift and the largest oscillator strength of the CAM-B3LYP absorption peaks when compared with the PBE0 or B3LYP ones have been observed in other complexes, while both PBE0 and B3LYP provide similar absorption spectra.^41–49^ In this study, the PBE0 calculated bands are in good agreement with the experimental values.

The main strong Soret peak of the Ni-TPP and Zn-TPP was calculated at 388 and 391 nm, in the DMF solvent, respectively. The corresponding vis absorption peaks of the **a** tautomers of the M-NCTPP present a red shift of about 22 nm, i.e., at 409 nm (Ni-NCTPP_a) and 414 nm (Zn-NCTPP_a), in very good agreement with the available experimental value of the 426 nm^62^ (Ni-NCTPP). The corresponding **b** tautomers of the M-NCTPP present larger red shifts than the **a** tautomers; i.e., the main vis absorption peaks are at 445 nm (Ni-NCTPP_b) and 425 nm (Zn-NCTPP_b). For the M-NCTPP-p complexes, strong Soret peaks are observed in the area 400–481 nm. Regarding the **a** tautomers of M-NCTPP-p, additional red shifts are observed with respect to the **a** tautomers of the M-NCPP. In the M-NCTPP-p_a complexes, the main vis absorption peaks are at 451 nm (Ni) and 481 nm (Zn) for the **a.cis** isomers and 444 nm (Ni) and 473 nm (Zn) for the **a.trans** isomers. In the M-NCTPP-p_b complexes, the main vis absorption peaks are at 475 nm (Ni) and 473 nm (Zn) for the **b.cis** isomers and 491 nm (Ni) and 493 nm (Zn) for the **b.trans** isomers. Finally, strong absorption peaks are observed in the area of 335–392 nm with oscillator strengths up to 0.77.

The plots of the absorption spectra of the M-TPP, M-NCTPP, and M-NCTPP-p complexes show the significant shifts of the absorption spectra of the various tautomers and isomers, Figure 4. Regarding the M-NCTPP, the absorption spectra of the **a** and **b** tautomers are distinct. The Q and Soret bands of **a** are significantly shifted with respect to the **b** tautomer. For the Ni^II^ cation, the distinction is clear for both bands; for the Zn^II^ cation, the distinction is clear for the Q-band, while for the Soret band, the main peaks are shifted by only 10 nm, see Figure 5i,ii. Regarding M-NCTPP-p, the absorption spectra of the different tautomers and trans–cis isomers differ. For both metals, the **b.trans** differs significantly from the other three structures. The **a.trans** and **a.cis** differ with respect to the intention of the main Soret peak. Finally, comparing the two metals, the Ni-NCTPP-p presents main Soret peaks below 450 nm, with the exception of the **b.trans** isomers, while the Zn-NCTPP-p presents main Soret peaks below 450 nm. Both metal complexes have a weak absorption peak at about 570 nm, while the Ni^II^ complex has an additional peak at about 650 nm. Thus, depending on the application of interest, the appropriate metal and its tautomer/isomer can be used with specific photophysical properties. Note that for the NCTPP, it has been found that different solvents can distinguish the tautomers and lead to the formation of different tautomers.^18^

### 3.4 De-Excitation

Selected Soret excited states of the eight M-NCTPP-p isomers that present a high oscillator strength and correspond to the main UV–vis absorption peaks were geometry-optimized. Their vertical de-excitation energies are given in Table 2 and depicted in Figure S4 of the Supporting Information. It should be noted that direct de-excitation from the Soret band to the ground state without passing through the S_1_ state is unusual. Usually, the emission pathway is Soret band → Q-band → S_0_ via a nonradiative decay or via fluorescence from the Q-band. However, after absorption in the Soret band, the complex could transit from the excited state back to the ground state in special occasions, even though the peak is very intense.^64,65^

**Table 2:** Energy Differences Δ*Ε* (eV), Wavelengths λ (nm), *f*-Values, and Corresponding cLR-Corrected Values of the De-Excitation Energies after Geometry Relaxation of the M-NCTPP-P Tautomers and Isomers at the PBE0/6-31G­(d,p) Level of Theory; the Corresponding Absorption Peaks λ_abs_ (nm) Are Also Included

M	complex	Λ	Δ*Ε*	*f*	λ_cLR_	Δ*Ε*_cLR_	λ_abs_
**Ni**	**a.cis**	478.4^a^	2.591	0.0592	486.4	2.549	454.3
		483.9	2.562	1.0984	465.8	2.662	451.3
	**a.trans**	507.2	2.445	0.9564	470.2	2.637	444.0
	**b.cis**	608.2	2.039	0.1672	597.5	2.075	474.8
	**b.trans**	584.2	2.122	0.3291	523.6	2.368	491.2
							
**Zn**	**a.cis**	559.1	2.217	1.4860	513.9	2.413	480.8
		424.4^a^	2.922	0.8331	411.9	3.010	368.4
	**a.trans**	558.5	2.220	1.7464	508.7	2.437	473.0
		419.9^a^	2.953	1.1739	399.2	3.106	389.8
	**b.cis**	592.9	2.091	1.2308	547.4	2.265	473.4
		456.1^a^	2.719	1.2002	427.6	2.899	399.5
	**b.trans**	589.9	2.102	1.4408	537.0	2.309	493.1
		517.5^a^	2.396	0.5391	502.3	2.468	461.7

a They have a CT or partial CT character.

In the present study, selected excited states of the M-NCTPP-p isomers that correspond to the main peaks in the vis area of the eight M-NCTPP-p isomers have been geometry-optimized. The de-excitation energies have been calculated as well as their cLR-corrected values. The last ones are shifted by 8–61 nm with respect to the uncorrected ones. These shifts are blue-shifted except for the peak at 478 nm of the Ni-NCTPP-p-a.cis, which is only −8 nm red-shifted. The λ_cLR_ de-excitation peaks are red-shifted with respect to the λ_abs_ values and the shifts range from 9 to 122 nm. For the Ni-NCTPP-p, the **a** tautomer presents strong de-excitation peaks at about 470 nm. All calculated tautomers/isomers of the Zn-NCTPP-p present strong peaks at about 530 nm, specifically at 509 nm (**a.trans**), 514 nm (**a.cis**), 537 nm (**b.trans**), and 547 nm (**b.cis**), see Figure S4 of the Supporting Information.

These de-excitation energies were calculated to investigate if they are close enough to the absorption S_0_ → Q bands, i.e., meaning that their decay energy can be absorbed by an adjacent complex. So, this can occur in the case of the Ni-NCTPP-p_b.cis, where the λ_cLR_ de-excitation peak is at 598 nm and the λ_abs_ peak of the first absorption peak is at 566 nm.

### 3.5 Frontier MOs

The frontier MOs involved in the main Q and Soret absorption peaks are given in Figures 6 and S3 of the Supporting Information. The main involved MO excitations for specific absorption peaks are noted. Their coefficients for the H → L excitation are ∼0.57 for the Ni^II^ complexes and ∼0.90 for the Zn^II^ complexes, while for the main absorption peaks, the coefficients are ∼0.61 for the Ni^II^ complexes and ∼0.71 for the Zn^II^ complexes. Both metals present a similar MO. The electron densities of the two lowest HOMO (H and H – 1) and LUMO (L and L + 1) orbitals are mainly in the porphyrin’s core. For both metal complexes, the H → L excitation is not a charge transfer (CT) transition for the M-TPP and M-NCTPP. On the contrary, in the case of the M-NCTPP-p isomers, the H → L corresponds to a partial CT excitation from the porphyrin’s core to peripheral group of ethenyl-pyrazine. Thus, the electron density in the LUMO is in both porphyrin’s core and the group of ethenyl-pyrazine, see Figures 6 and S3 of the Supporting Information. Furthermore, the absorption peaks in the UV–vis area, around 340–380 nm, also correspond to partial CT excitations, see Figure 7 for the Zn-NCTTP-p_a.cis at 368 nm; the coefficient of this configuration is 0.707. Generally, the L– 1 → H or L → H + 2 or L → H + 3 transitions can be characterized as partial CT transitions. Finally, it should be noted that Gouterman’s model, a simple model which explains the absorption spectra of the plain porphyrin, presents deviations in the cases of M-NCTPP and M-NCTPP, which are increased when the ethenyl-pyrazine group is added.

**Figure 6:**
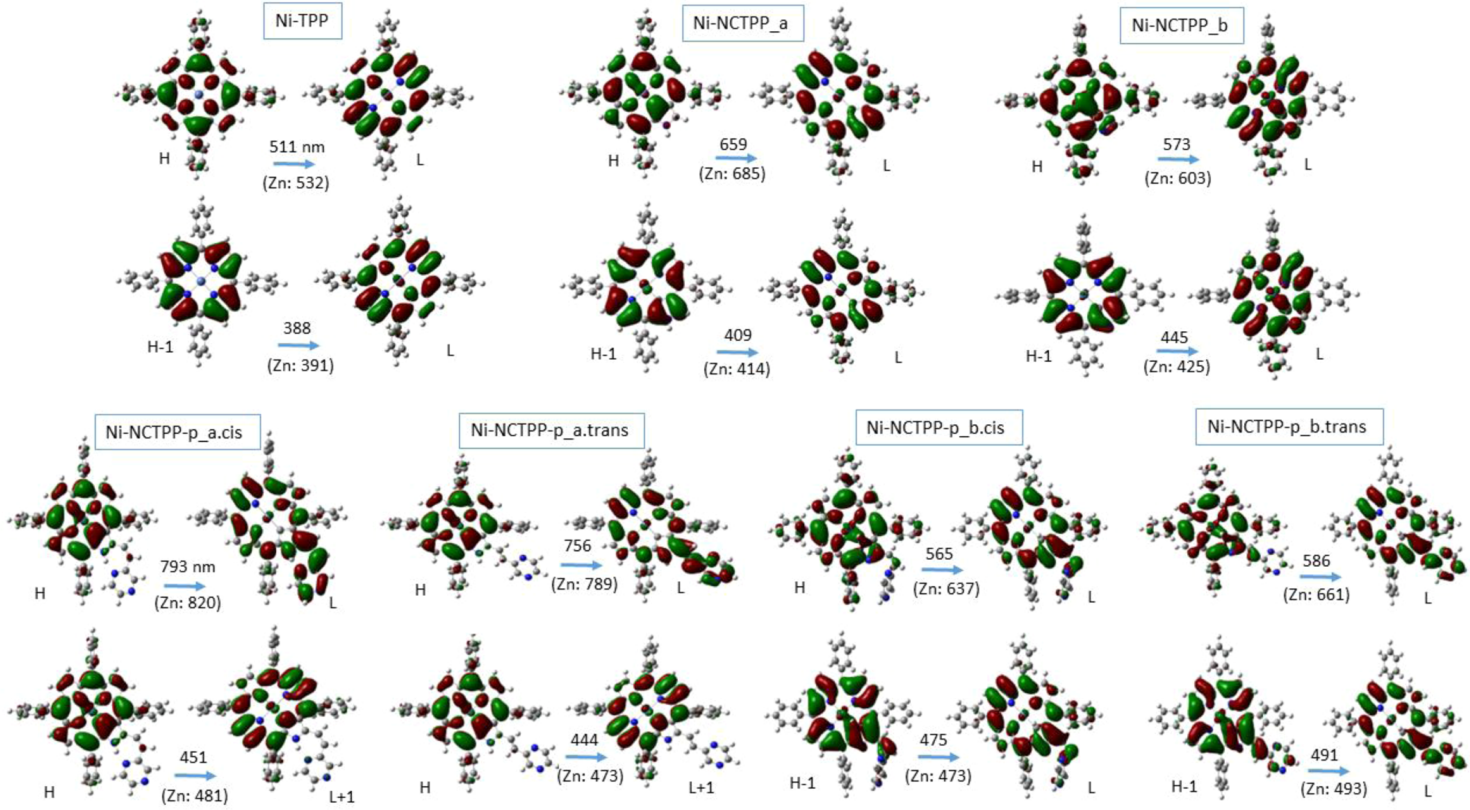
Frontier MOs correspond to the H → L and main absorption peaks of Ni^II^ complexes. In parentheses are given the corresponding values of the Zn^II^ complexes.

**Figure 7:**
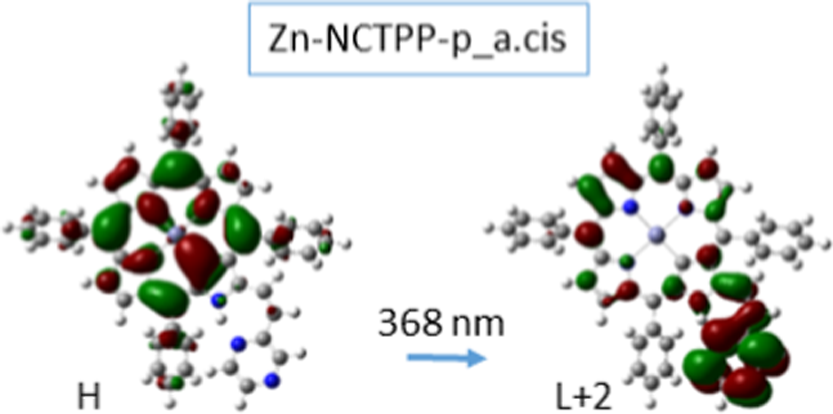
CT excitation of the Zn-NCTPP-p_a.cis isomer.

Overall, the selection of the metal and the peripheral group lead to different lowest in energy structure, affect its absorption spectrum, and thus they tune the photophysical properties of the M-NCP complexes. Generally, the tautomers of NCP are interconvertible^20^ and can be separated.^18^ Thus, since the present calculated structures and their electronic properties differ, specific tautomers of the calculated complexes may be used as candidates for applications such as optical memories, switches, and molecular logic gates depending on the desired photophysical properties. The NCPs can be physically or chemically attached in two-dimensional (2D) materials and be involved in electron transfer processes from the surface to the NCPs and vice versa.^66,67^ Tuning their spectra, they can be combined with appropriate 2D materials having specific properties.

## 4 Summary and Conclusions

In the present study, metal complexes of Zn^II^ and Ni^II^ of TPP, NCTPP, and ethenyl-pyrazine derivatives of NCTPP, i.e., M-TPP, M-NCTPP, and M-NCTPP-p, have been calculated via DFT and TD-DFT calculations. The photophysical properties of the molecules, their structures (tautomers, trans and cis isomers), their absorption spectra, and de-excitation energies have been studied.

In the M-NCTPP and M-NCTPP-p complexes, the **b** tautomer, which has an H atom in the porphyrin core, has a more deformed core than the core of the **a** tautomer, where the H atom is bonded to the N atom of the reversed pyrrolium ring. In the M-NCTPP-p complexes, the cis and trans isomerization is a result of the pyrazine group. The **a.cis** isomers are more stable than **a.trans** for both metals by about 1.3 kcal/mol, while the energy ordering of trans/cis isomers of the **b** tautomer follow the opposite ordering, i.e., **b.trans** are more stable than **b.cis** for both metals by about 8.5 kcal/mol. Comparing the two metals, the Ni^II^ and Zn^II^ complexes of the M-NCTPP and Ni-NCTPP-p have different lowest energy structures, i.e., the Ni-NCTPP_a, Zn-NCTPP_b, Ni-NCTPP-p_a.cis, and Zn-NCTPP-p_b.trans structures.

Regarding the UV–vis absorption spectra, plain TPPs have absorption spectra with one main peak, while NCTPPs have two main peaks. Adding the ethenyl-pyrazine group in NCTPP, additional peaks are added to the UV–vis absorption spectra. The addition of the pyrazine group results in red shifts of the absorption spectra, i.e., from the area of 380–440 nm to 430–500 nm. The **a.trans** isomers present the lowest in energy located peaks, i.e., the most red-shifted peaks. The absorption main peaks of M-NCTPP-p are red-shifted compared to M-NCTPP up to 80(135) nm for the Soret­(Q) bands. The different isomers present shifts of the main absorption peaks up to 50(180) nm.

Finally, regarding the de-excitation energies, the fluorescent peaks present shifts up to 50 nm depending on the isomer. It is found that the vertical de-excitation energy of the Soret peak of the Ni-NCTPP-p_b.cis complex to the S_0_ has λ_cLR_ = 598 nm, while the λ_abs_ peak of the first absorption peak is at 566 nm.

For both metal complexes, the H → L excitation is not a CT transition for the M-TPP and M-NCTPP. On the contrary, in the case of the M-NCTPP-p isomers, the H → L corresponds to a partial CT excitation from the porphyrin’s core to peripheral group of ethenyl-pyrazine, and the electron density is in both the porphyrin’s core and the group of ethenyl-pyrazine. Furthermore, the absorption peaks in the UV–vis area of around 340–380 nm also correspond to partial CT excitations. Finally, comparing the simple Gouterman’s model with our results, it presents deviations in the cases of the M-NCTPP and M-NCTPP-p, which are larger in the last complexes, where the ethenyl-pyrazine group is added. This is expected to happen since both M-NCTPP and M-NCTPP-p are not as symmetric as the simple porphyrin metal complexes.

To sum up, the selection of the peripheral group and of the metal results in different lowest energy structure and influences its absorption spectrum. Thus, the photophysical properties of the M-NCTPP complexes can be tuned. Given that the tautomers are, in general, interconvertible,^68^ and have different electronic properties, they may have applications such as optical memories, switches, sensors and molecular logic gates.^7,68,69^

## Supplementary Material



## Data Availability

The data supporting this article have been included as part of the Supporting Information.

## Notes

Geometries, relative energies, population analysis, excitation energies, and MOs of the calculated structures of the M-TPP, M-NCTPP, and M-NCTPP-p complexes (PDF)
