# Supplementary material for: Tuning the Photophysical Properties of Nickel and Zinc Complexes of N‑Confused Tetraphenylporphyrin via Trans–Cis Isomerization
Source: J Phys Chem A. 2025 Jun 26;129(27):5942–52. doi: 10.1021/acs.jpca.5c02035 (PMC12257541; doi:10.1021/acs.jpca.5c02035)
Supplement: Supplementary file 1 [file jp5c02035_si_001.pdf]

Supporting Information

Tuning the Photophysical Properties of Nickel and Zinc Complexes of N-Confused Tetraphenylporphyrin via trans-cis Isomerization

Eleftherios Papamichalis,[a] Ioannis D. Petsalakis,[b] and Demeter Tzeli[a,b*]

[a] *Laboratory of Physical Chemistry, Department of Chemistry, National and Kapodistrian University of Athens, Panepistimiopolis Zografou, Athens 157 84, Greece*

[b] *Theoretical and Physical Chemistry Institute, National Hellenic Research Foundation, 48 Vassileos Constantinou Ave., Athens 116 35, Greece*

E-mail: tzeli@chem.uoa.gr

Tel: +30-210-727-4307

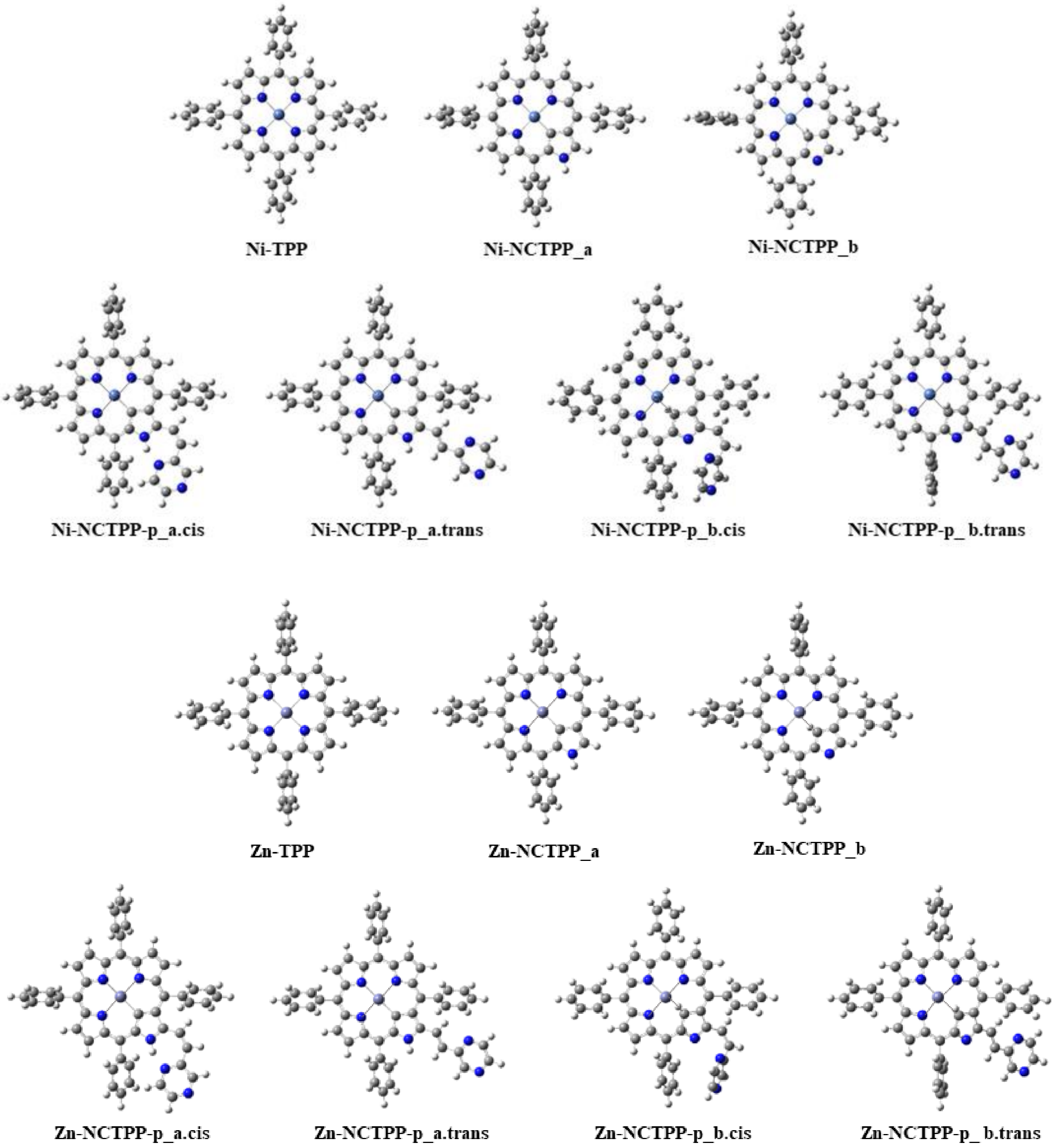

Figure S1. Metal complexes of tetraphenylporphyrin, M-TPP, N-confused tetraphenylporphyrin, M-NCTPP (M-NCTPP_a and M-NCTPP_b tautomers) and ethenyl-pyrazine derivative M-NCTPP-p (M-NCTPP-p_a.trans , M-NCTPP-p_a.cis, M-NCTPP-p_b.trans and M-NCTPP-p_b.cis).

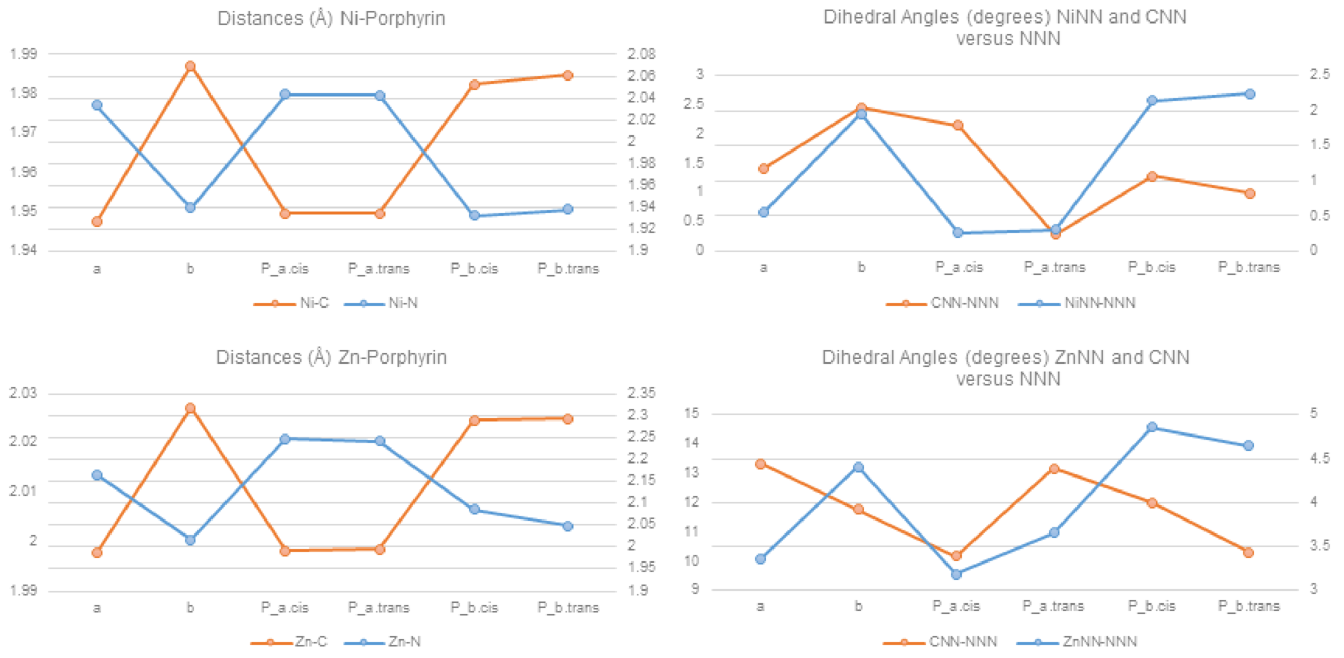

Figure S2. (i) Left column: Bond distance between Metal and nearest nitrogen and carbon atom of the porphyrin core **(ii)** Right column NNNM and NNNC dihedral angles of Porphyrin's core

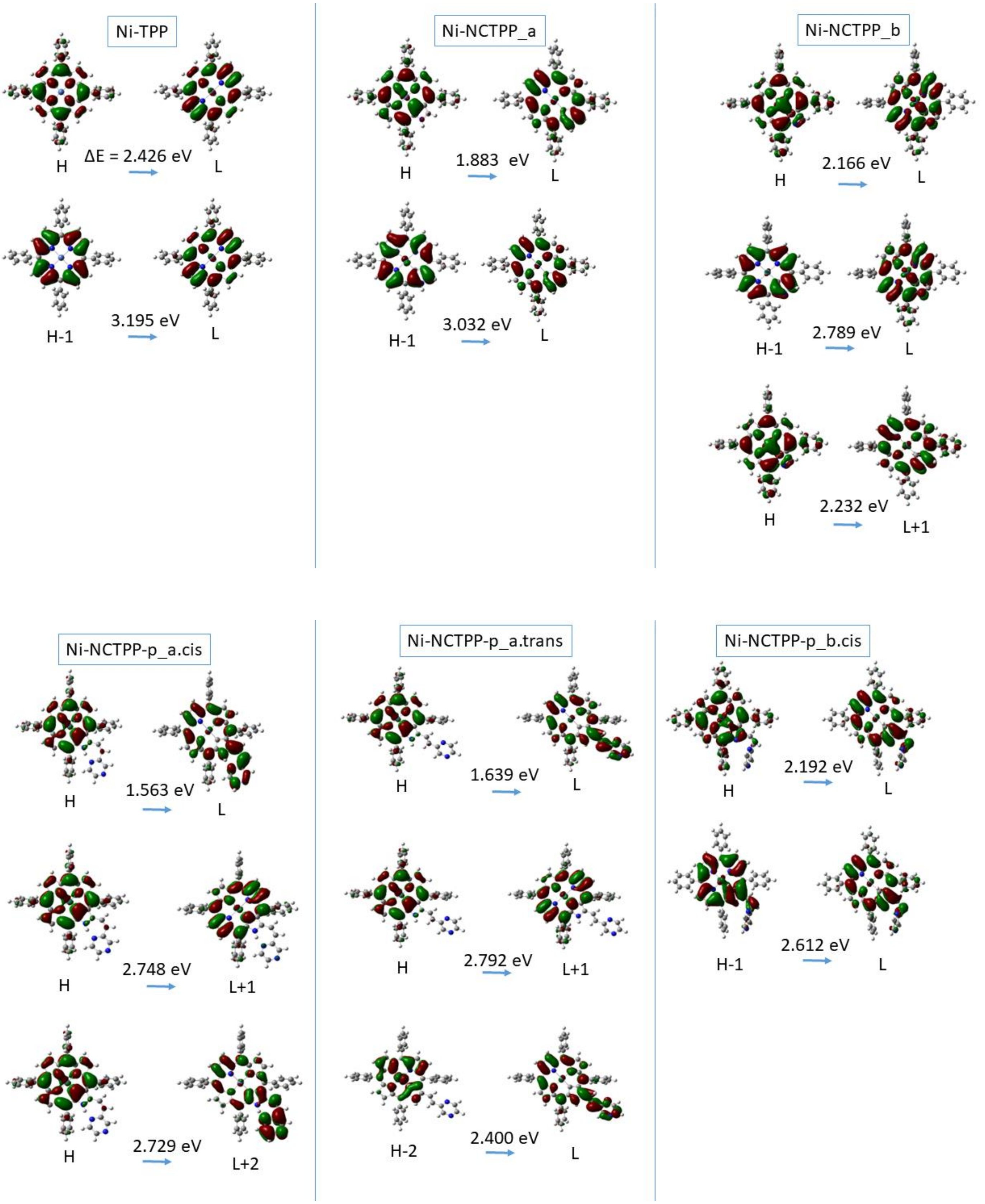
Ni-TPP
H
ΔE = 2.426 eV
L
H-1
3.195 eV
L
Ni-NCTPP_a
H
1.883 eV
L
H-1
3.032 eV
L
Ni-NCTPP_b
H
2.166 eV
L
H-1
2.789 eV
L
H
2.232 eV
L+1
Ni-NCTPP-p_a.cis
H
1.563 eV
L
H
2.748 eV
L+1
H
2.729 eV
L+2
Ni-NCTPP-p_a.trans
H
1.639 eV
L
H
2.792 eV
L+1
H-2
2.400 eV
L
Ni-NCTPP-p_b.cis
H
2.192 eV
L
H-1
2.612 eV
L

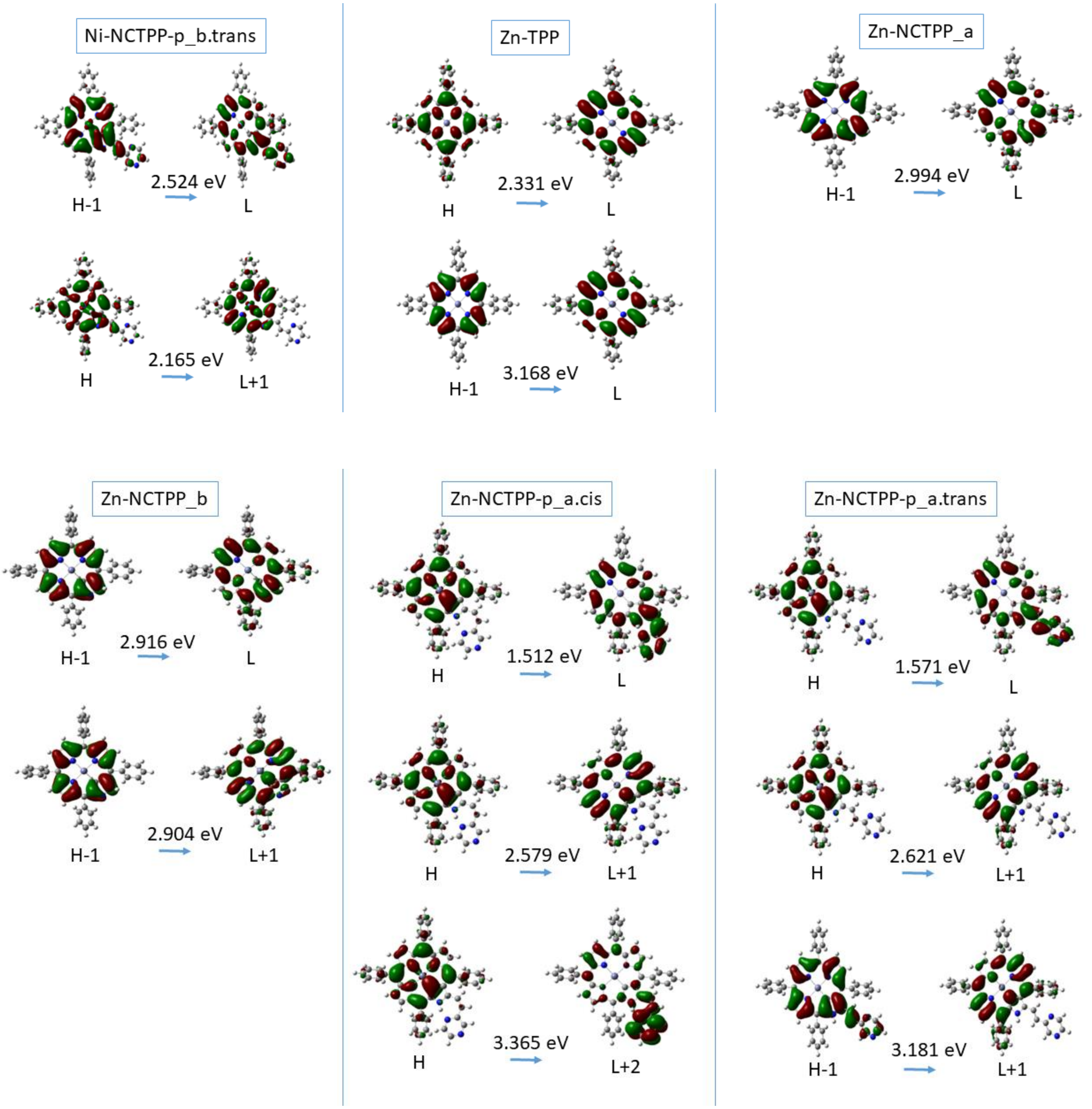
Ni-NCTPP-p_b.trans
H-1
2.524 eV
L
H
2.165 eV
L+1
Zn-TPP
H
2.331 eV
L
H-1
3.168 eV
L
Zn-NCTPP_a
H-1
2.994 eV
L
Zn-NCTPP_b
H-1
2.916 eV
L
H-1
2.904 eV
L+1
Zn-NCTPP-p_a.cis
H
1.512 eV
L
H
2.579 eV
L+1
H
3.365 eV
L+2
Zn-NCTPP-p_a.trans
H
1.571 eV
L
H
2.621 eV
L+1
H-1
3.181 eV
L+1

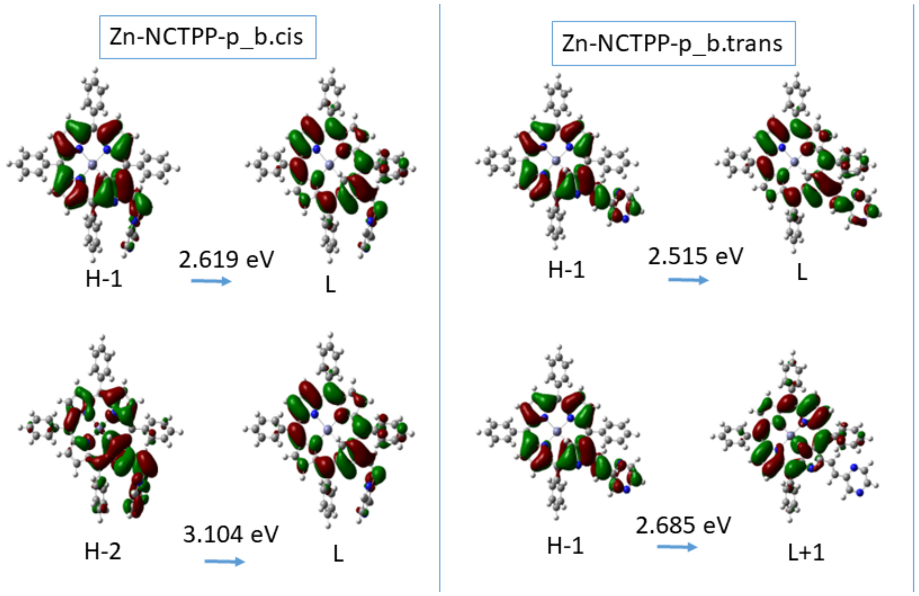

Figure S3. Main molecular orbitals involved in absorption UV-vis peaks of tetraphenylporphyrin, M-TPP, N-confused tetraphenylporphyrin, M-NCTPP (M-NCTPP_a and M-NCTPP_b tautomers) and ethenyl-pyrazine derivative M-NCTPP-p (M-NCTPP-p_a.trans , M-NCTPP-p_a.cis, M-NCTPP-p_b.trans and M-NCTPP-p_b.cis). The corresponding ΔE energies of the main absorption peaks are given.

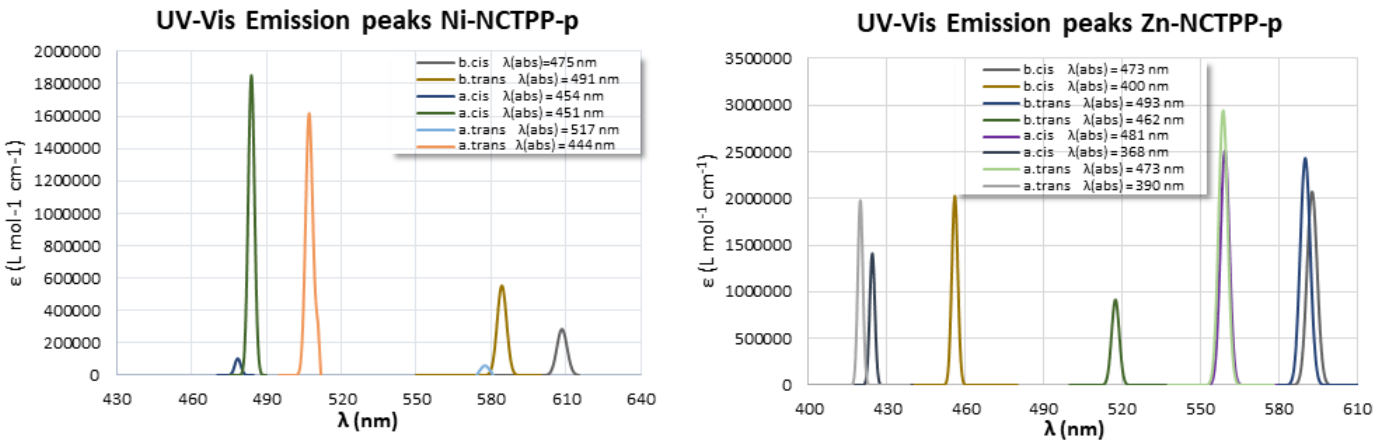

Figure S4 Vertical de-excitation energies of the M-NCTPP and M-NCTPP-p tautomers and isomers at the PBE0/6-31G(d,p) level of theory in DMF solvent.

Table S1. Relative energy differences in kcal/mol of calculated minima structures at the PBE0/6-31G(d,p) methodology in DMF.

**Complex**	**Ni**	**Zn**
**M-TPP**	0.00	0.00
**M-NCTPP_a**	20.15	32.82
**M-NCTPP_b**	29.00	26.61
**M-NCTPP_a.cis**	0.00	4.62
**M-NCTPP_a.trans**	1.49	5.77
**M-NCTPP_b.cis**	16.47	8.93
**M-NCTPP_b.trans**	8.09	0.00

Table S2. N-M and C-M bond distance in Å, between the metal and nitrogen[a] or carbon atom of the porphyrin core of the calculated minima structures at the PBE0/6-31G(d,p) methodology in DMF.

**Complex**	**N-Ni**	**C-Ni**	**N-Zn**	**C-Zn**
**M-NCTPP_a**	1.977	1.927	2.013	1.986
**M-NCTPP_b**	1.951	2.070	2.000	2.317
**M-NCTPP_a.cis**	1.980	1.935	2.021	1.992
**M-NCTPP_a.trans**	1.980	1.935	2.020	1.994
**M-NCTPP_b.cis**	1.949	2.053	2.006	2.289
**M-NCTPP_b.trans**	1.951	2.061	2.003	2.292

[a] The shortest M-N bond distance is given.

Table S3. NNNM and NNNC dihedral angles in degrees of the porphyrin's core of the calculated minima structures at the PBE0/6-31G(d,p) methodology in DMF.

**Complex**	**NNNNi**	**NNCNi**	**NNNZn**	**NNCZn**
**M-NCTPP_a**	0.674	1.185	10.089	4.441
**M-NCTPP_b**	2.338	2.044	13.232	3.923
**M-NCTPP_a.cis**	0.319	1.787	9.547	3.393
**M-NCTPP_a.trans**	0.370	0.237	10.972	4.390
**M-NCTPP_b.cis**	2.567	1.072	14.584	4.002
**M-NCTPP_b.trans**	2.692	0.826	13.933	3.433

Table S4. Selected geometries calculated via PBE0/6-31G(d,p) and PBE0-D3/6-31G(d,p) levels of theory in DMF.

Methodology	M-N	M-N	M-N	M-C	C-H	CNNN	MNNN	MCH
**Ni-NCTPP-p_b.cis**								
PBE0/6-31G(d,p)	1.949	1.979	1.982	2.053	1.101	1.07	2.57	72.32
PBE0-D3/6-31G(d,p)	1.924	1.940	1.942	1.969	1.109	2.51	3.01	72.30
**Zn-NCTPP-p_a.trans**								
PBE0/6-31G(d,p)	2.020	2.126	2.134	1.995		4.39	10.97	
PBE0-D3/6-31G(d,p)	2.005	2.119	2.132	1.962		4.41	10.15	

Table S5. Calculated Mulliken, CM5, and NBO charges on M, where M= Ni and Zn at the PBE0/6-31G(d,p) level of theory in DMF.

	Q_M			Metal Valence Electron charges
	**Mulliken**	**CM5**	**NPA**	**NPA**
**Ni-NCTPP_b**	+0.80	+0.53	+0.94	[core]4S(0.39)3d(8.36)4p(0.31)5S(0.01)
**Ni-NCTPP-p_b.cis**	+0.79	+0.52	+0.92	[core]4S(0.39)3d(8.37)4p(0.31)5S(0.01)
**Ni-NCTPP-p_a.trans**	+0.71	+0.47	+0.84	[core]4S(0.45)3d(8.34)4p(0.37)5S(0.01)
**Zn-NCTPP_b**	+1.10	+0.82	+1.42	[core]4S(0.37)3d(9.91)4p(0.29)
**Zn-NCTPP-p_b.cis**	+1.10	+0.82	+1.42	[core]4S(0.37)3d(9.91)4p(0.29)
**Zn-NCTPP-p_a.trans**	+0.90	+0.67	+1.26	[core]4S(0.46)3d(9.90)4p(0.39)

Table S6. Energy differences ΔE (eV), wavelengths λ (nm), f-values of the main vis-UV absorption peaks of the Q and Soret bands of the M-NCTPP-p tautomers at the PBE0 and CAM-B3LYP/6-31G(d,p) levels of theory in DMF. Experimental values in italics.

	**λ**	**ΔE**	**f**		**λ**	**ΔE**	**f**		**λ**	**ΔE**	**f**		**λ**	**ΔE**	**f**
	**Ni-NCTPP-p_b.cis**								**Zn-NCTPP-p_a.trans**						
	**PBE0**				**CAM-B3LYP**				**PBE0**				**CAM-B3LYP**		
	565.6	2.192	0.037		562.3	2.205	0.045		789.4	1.571	0.115		710.4	1.745	0.139
	474.8	2.611	0.535		440.1	2.817	0.975		567.3	2.186	0.171		524.4	2.364	0.231
	455.3	2.723	0.388		415.3	2.986	1.053		473.0	2.621	1.273		437.0	2.837	1.918
	438.8	2.826	0.386												
Q[a]	*591*	*2.10*													
S[a]	*467*	*2.65*													

[a] Experimental main peaks at Q and Soret bands for the Ni-NCTPP, references 56-57.

Geometries

Methodology PBE0/6-31G(d,p) in DMF solvent

Ni-TPP

N	-0.0008	-1.9848	0.00001
N	-1.97255	-0.00638	-0.01146
N	0.0008	1.96138	-0.00024
N	1.97256	-0.00803	0.0115
C	1.08214	-2.82158	0.12331
C	0.66829	-4.20129	0.09403
C	-0.67164	-4.20077	-0.0939
C	-1.08442	-2.82072	-0.12324
C	-2.41683	-2.42449	-0.20962
C	-2.80843	-1.09064	-0.11093
C	-4.18662	-0.68798	0.00605
C	-4.18469	0.65223	0.19354
C	-2.80722	1.07245	0.15785
C	-2.41111	2.40407	0.25585
C	-1.07881	2.79749	0.1453
C	-0.66857	4.17718	0.09286
C	0.67198	4.1766	-0.09415
C	1.0811	2.79657	-0.14602
C	2.4131	2.40203	-0.25608
C	2.8081	1.07013	-0.15765
C	4.18526	0.6489	-0.1929
C	4.18612	-0.69132	-0.00544
C	2.80758	-1.09293	0.11121
C	2.41486	-2.42647	0.20981
H	1.32929	-5.04926	0.19004
H	-1.33328	-5.04825	-0.18986
H	-5.0312	-1.36013	-0.02133
H	-5.02864	1.30763	0.34715
H	-1.33341	5.02366	0.17753
H	1.33751	5.0225	-0.17919
H	5.02973	1.30371	-0.34618
H	5.03017	-1.36411	0.02224
Ni	-0.00001	-0.0099	-0.00004
C	3.47553	-3.46666	0.35214
C	3.72333	-4.38424	-0.67472
C	4.2532	-3.52341	1.51373
C	4.72286	-5.34396	-0.53919
H	3.13018	-4.3372	-1.58345
C	5.25344	-4.48275	1.64898
H	4.06708	-2.8107	2.31199
C	5.48942	-5.39588	0.62338
H	4.90621	-6.04752	-1.34474
H	5.84658	-4.51778	2.55698
H	6.26885	-6.14342	0.7286
C	3.45466	3.45556	-0.43442
C	4.39515	3.7071	0.57055
C	3.49622	4.21713	-1.60732
C	5.36073	4.6962	0.40386
H	4.36172	3.12439	1.48659
C	4.46161	5.20678	-1.77378
H	2.76745	4.02651	-2.38989
C	5.39637	5.44779	-0.76898
H	6.08198	4.8827	1.19287
H	4.48476	5.78747	-2.69017
H	6.14852	6.21904	-0.89872
C	-3.4518	3.45843	0.43435
C	-3.49198	4.22071	1.60684
C	-4.39295	3.70997	-0.57
C	-4.45667	5.21102	1.77352
H	-2.76271	4.03011	2.38895
C	-5.35784	4.69971	-0.4031
H	-4.3606	3.12673	-1.48575
C	-5.39211	5.45198	0.76935
H	-4.47874	5.79224	2.6896
H	-6.07963	4.88616	-1.19163
H	-6.14372	6.22373	0.89925
C	-3.47837	-3.4638	-0.35204
C	-4.25609	-3.51971	-1.51363
C	-3.72689	-4.38136	0.67466
C	-5.25706	-4.47827	-1.64906
H	-4.06943	-2.80698	-2.31174
C	-4.72716	-5.34029	0.53894
H	-3.13372	-4.33494	1.58341
C	-5.49373	-5.39143	-0.62365
H	-5.85023	-4.51268	-2.55708
H	-4.91106	-6.04386	1.34437
H	-6.27373	-6.13835	-0.72901

Ni-NCTPP_a

C	1.24623	2.79051	-0.01104
C	0.87768	4.18449	-0.01649
C	-0.47216	4.23682	0.01656
C	-0.9393	2.86884	0.01374
C	-1.24527	-2.76044	-0.03869
C	-0.92221	-4.16653	-0.02216
C	0.42866	-4.25257	0.01008
C	0.93254	-2.90515	0.02788
C	2.28368	-2.59384	0.03563
C	2.68641	-1.24942	-0.01509
C	4.15066	0.42573	0.03783
H	5.10886	0.92186	0.10186
C	1.89398	-0.10025	-0.07598
C	2.84163	0.96086	-0.05007
C	2.55384	2.34832	-0.03342
C	3.30671	-3.6706	0.11015
C	3.67779	3.32233	-0.03186
C	3.84257	4.23766	1.01517
C	4.91171	5.129	1.01299
C	5.83085	5.11814	-0.03441
C	5.67812	4.20883	-1.07901
C	4.61145	3.31449	-1.07634
H	1.57499	5.00864	-0.04624
H	-1.10716	5.1094	0.03329
H	-1.64308	-4.96981	-0.03561
H	1.04001	-5.14307	0.0128
H	3.13222	4.23776	1.83653
H	5.02924	5.82922	1.83355
H	6.66338	5.81396	-0.03539
H	6.38778	4.19711	-1.89978
H	4.48739	2.61129	-1.89484
N	0.09901	1.9906	0.0003
N	-0.11837	-1.99421	-0.0067
N	4.04533	-0.8938	0.05531
H	4.81692	-1.54426	0.12953
C	3.33839	-4.55261	1.19776
H	2.59963	-4.4482	1.98657
C	4.27384	-3.79895	-0.89594
C	4.31097	-5.54471	1.27324
H	4.3254	-6.21956	2.12273
C	5.26712	-5.66702	0.26643
H	6.02554	-6.44043	0.32729
C	5.24657	-4.79255	-0.81771
H	5.98404	-4.88586	-1.60803
H	4.24768	-3.12891	-1.75107
C	-2.57342	-2.28825	-0.06219
C	-2.89335	-0.94281	-0.03663
C	-3.68031	-3.28923	-0.11096
C	-4.55493	-3.43913	0.97074
C	-3.86452	-4.09205	-1.24261
C	-5.59301	-4.36652	0.92126
H	-4.41486	-2.82211	1.85381
C	-4.89954	-5.02188	-1.2919
H	-3.19025	-3.98	-2.08703
C	-5.76731	-5.16057	-0.21008
H	-6.26265	-4.47116	1.76885
H	-5.03149	-5.63455	-2.17792
H	-6.57506	-5.88412	-0.24894
C	-4.25981	-0.44629	-0.08967
C	-4.18746	0.89707	-0.05524
C	-2.77513	1.23949	0.01189
N	-2.00422	0.10286	0.0133
H	-5.13992	-1.06819	-0.15942
H	-4.99477	1.61344	-0.09126
C	-2.31201	2.54204	0.02436
C	-3.30754	3.65506	0.03963
C	-3.44603	4.50552	-1.06334
C	-4.12432	3.86229	1.15649
C	-4.37612	5.54133	-1.04827
H	-2.81826	4.34803	-1.93595
C	-5.05798	4.89588	1.17139
H	-4.02099	3.20649	2.01638
C	-5.18477	5.73895	0.06968
H	-4.47297	6.19098	-1.91218
H	-5.68299	5.04456	2.04608
H	-5.91041	6.54567	0.08142
Ni	-0.03	-0.00036	-0.01947

Ni-NCTPP_b

C	-1.14268	2.81553	0.29002
C	-0.7229	4.17873	0.46877
C	0.62963	4.19083	0.38448
C	1.04238	2.8279	0.18083
C	1.12324	-2.78308	0.20551
C	0.74069	-4.15949	0.40193
C	-0.61107	-4.1866	0.4342
C	-1.06684	-2.83265	0.23487
C	-2.41987	-2.50056	0.08434
C	-2.80197	-1.15759	0.02676
C	-4.03372	0.54377	-0.45515
H	-4.86134	1.11611	-0.86102
C	-2.00104	-0.0351	0.40657
C	-2.82923	1.0899	0.09677
C	-2.48957	2.4369	0.17269
C	-3.44389	-3.56156	-0.08034
C	-3.55691	3.46062	0.04518
C	-3.46202	4.50245	-0.88819
C	-4.48925	5.43133	-1.01789
C	-5.62726	5.33388	-0.21874
C	-5.73633	4.29815	0.70666
C	-4.71089	3.3659	0.83483
H	-1.38764	5.01305	0.63564
H	1.29596	5.03737	0.45337
H	1.42906	-4.98536	0.50026
H	-1.2576	-5.03906	0.57862
H	-1.48364	-0.06618	1.37697
H	-2.58535	4.56793	-1.52527
H	-4.40456	6.2275	-1.75018
H	-6.42709	6.06007	-0.32001
H	-6.6191	4.21662	1.33224
H	-4.79066	2.56273	1.56137
N	-0.04173	1.98771	0.14382
N	0.01533	-1.97559	0.12746
N	-4.0182	-0.77134	-0.50263
C	-3.2828	-4.59056	-1.01727
H	-2.39485	-4.60678	-1.64219
C	-4.61087	-3.53353	0.69473
C	-4.2568	-5.57422	-1.16483
H	-4.11958	-6.36089	-1.89957
C	-5.40606	-5.54382	-0.37813
H	-6.1642	-6.3119	-0.49147
C	-5.58098	-4.51932	0.55136
H	-6.47457	-4.48915	1.16634
H	-4.74593	-2.73404	1.41622
C	2.45075	-2.37941	0.05299
C	2.82837	-1.04595	-0.1061
C	3.52126	-3.42105	0.05565
C	4.20873	-3.73173	-1.12205
C	3.85592	-4.09352	1.23552
C	5.21326	-4.6962	-1.11969
H	3.95026	-3.21268	-2.04063
C	4.85783	-5.06063	1.23679
H	3.32687	-3.85203	2.15302
C	5.53905	-5.36325	0.05946
H	5.73823	-4.92909	-2.04041
H	5.10924	-5.57418	2.15908
H	6.32093	-6.11558	0.06103
C	4.19611	-0.61222	-0.26834
C	4.17574	0.74066	-0.29037
C	2.7963	1.13807	-0.13814
N	1.98949	0.03252	-0.03533
H	5.04979	-1.27003	-0.33523
H	5.0091	1.42159	-0.37962
C	2.38091	2.45786	-0.00272
C	3.41791	3.53185	-0.04812
C	3.76024	4.24046	1.10822
C	4.06661	3.83551	-1.24919
C	4.73081	5.23781	1.06266
H	3.26204	4.00361	2.04398
C	5.03922	4.83131	-1.29364
H	3.80281	3.28706	-2.14889
C	5.37234	5.53503	-0.13822
H	4.98899	5.77935	1.96688
H	5.53392	5.0591	-2.2322
H	6.12932	6.31161	-0.17316
Ni	0.04176	0.00413	0.07477

Ni-NCTPP-p_a.trans

C	-0.54915	2.4864	-0.73516
C	-0.38834	3.88242	-1.03838
C	0.91403	4.18806	-0.81646
C	1.55416	2.97502	-0.38287
C	2.79849	-2.47212	-0.08169
C	2.75629	-3.89545	-0.32707
C	1.46959	-4.2075	-0.5941
C	0.71117	-2.97947	-0.52271
C	-0.68287	-2.95087	-0.57233
C	-1.35973	-1.72584	-0.47085
C	-2.98144	-0.34381	-0.08616
C	-0.78562	-0.44488	-0.74408
C	-1.86978	0.46552	-0.53027
C	-1.79823	1.84629	-0.71056
C	-1.45302	-4.2211	-0.68374
C	-3.03805	2.64785	-0.86472
C	-3.97965	2.28531	-1.83662
C	-5.17862	2.98092	-1.95173
C	-5.45513	4.04273	-1.09171
C	-4.52357	4.41267	-0.12324
C	-3.31899	3.72364	-0.01315
H	-1.17698	4.53424	-1.38375
H	1.40804	5.13964	-0.94287
H	3.6099	-4.5553	-0.28914
H	1.04934	-5.17957	-0.8036
H	-3.76492	1.44968	-2.49629
H	-5.89906	2.692	-2.70983
H	-6.39473	4.57911	-1.17465
H	-4.73732	5.23416	0.55256
H	-2.60002	3.99763	0.75312
N	0.66783	1.93059	-0.37537
N	1.55476	-1.91658	-0.23158
N	-2.66371	-1.63619	-0.07862
C	-1.35681	-5.00075	-1.8416
H	-0.71849	-4.66782	-2.65522
C	-2.28826	-4.64884	0.35435
C	-2.07305	-6.18975	-1.95697
H	-1.98832	-6.78324	-2.86156
C	-2.89752	-6.61137	-0.91675
H	-3.45611	-7.5375	-1.00496
C	-3.00323	-5.83728	0.23774
H	-3.64276	-6.16119	1.05259
H	-2.37324	-4.04222	1.24929
C	3.96464	-1.79995	0.29467
C	4.0241	-0.4116	0.43346
C	5.19848	-2.59403	0.55868
C	6.35275	-2.39008	-0.20667
C	5.22463	-3.55253	1.57868
C	7.50638	-3.12876	0.0412
H	6.33743	-1.6517	-1.00317
C	6.37842	-4.29067	1.82708
H	4.3345	-3.71105	2.18067
C	7.52191	-4.08041	1.05895
H	8.39178	-2.96331	-0.56392
H	6.38513	-5.02685	2.62424
H	8.4211	-4.65605	1.25247
C	5.18517	0.30575	0.90792
C	4.88995	1.62372	0.82496
C	3.54669	1.71767	0.30259
N	3.02686	0.46222	0.09966
H	6.09432	-0.15445	1.26501
H	5.50666	2.46557	1.10236
C	2.90121	2.91365	0.01006
C	3.66851	4.18552	0.1245
C	3.2472	5.19519	0.99846
C	4.82547	4.38928	-0.63703
C	3.9683	6.38029	1.11006
H	2.35389	5.04085	1.59657
C	5.54481	5.57608	-0.52729
H	5.15304	3.61312	-1.32249
C	5.11836	6.57365	0.34699
H	3.63376	7.15117	1.79649
H	6.43638	5.72242	-1.12818
H	5.67969	7.49822	0.43332
C	-4.27942	0.1095	0.40359
H	-4.42515	1.16927	0.57917
C	-5.298	-0.73092	0.64424
H	-5.17185	-1.79686	0.47248
C	-6.6068	-0.28187	1.12456
C	-7.6218	-1.21439	1.38544
H	-7.438	-2.27553	1.23448
C	-8.02984	1.38444	1.73924
N	-6.82508	1.03123	1.30578
N	-8.82929	-0.85962	1.81994
C	-9.03195	0.44676	1.99706
H	-10.00945	0.76136	2.34996
H	-8.21536	2.44483	1.88738
H	-0.19658	-0.34123	-1.669
Ni	1.15581	0.01873	-0.22843

Ni-NCTPP-p_a.cis

C	-0.30083	-2.90603	-0.36492
C	-0.94012	-4.15052	-0.69226
C	-2.27829	-3.9394	-0.62035
C	-2.45893	-2.55952	-0.25884
C	-1.59477	2.97264	-0.13634
C	-0.98854	4.2633	-0.37186
C	0.34494	4.06015	-0.44983
C	0.56899	2.64438	-0.27226
C	1.84166	2.09085	-0.12672
C	1.97791	0.70745	0.05222
C	2.92255	-1.16665	0.58009
C	1.0078	-0.27428	-0.3184
C	1.64666	-1.51632	0.00361
C	1.08863	-2.77636	-0.19261
C	3.05905	2.94994	-0.15009
C	1.95623	-3.97971	-0.2055
C	3.09091	-4.00117	-1.0276
C	3.94231	-5.10092	-1.02832
C	3.67778	-6.18925	-0.19884
C	2.55586	-6.17524	0.6285
C	1.69738	-5.07992	0.62313
H	-0.42244	-5.06205	-0.95149
H	-3.07648	-4.64188	-0.80737
H	-1.52853	5.1943	-0.45662
H	1.12435	4.79316	-0.59481
H	3.3019	-3.14473	-1.66171
H	4.81435	-5.10698	-1.67388
H	4.34531	-7.04469	-0.19428
H	2.35168	-7.01568	1.28363
H	0.83246	-5.06038	1.2793
N	-1.25131	-1.92218	-0.14474
N	-0.64155	1.98761	-0.10599
N	3.09496	0.14863	0.60404
C	3.40074	3.64802	-1.31417
H	2.75833	3.57286	-2.18712
C	3.90382	3.03624	0.96305
C	4.55726	4.42338	-1.36316
H	4.80845	4.95727	-2.27401
C	5.39009	4.50526	-0.2502
H	6.294	5.10479	-0.28805
C	5.05985	3.80866	0.91167
H	5.70828	3.8625	1.78038
H	3.65323	2.48305	1.86124
C	-2.96549	2.80046	0.07156
C	-3.55691	1.54163	0.21014
C	-3.83969	4.00542	0.15015
C	-4.88114	4.18555	-0.76805
C	-3.64046	4.97167	1.14346
C	-5.70286	5.30677	-0.6965
H	-5.03834	3.4418	-1.54388
C	-4.46252	6.09292	1.21533
H	-2.83952	4.83436	1.8642
C	-5.4953	6.2632	0.29545
H	-6.50269	5.43502	-1.4185
H	-4.29891	6.83103	1.99373
H	-6.13581	7.13723	0.35171
C	-4.94797	1.33349	0.53911
C	-5.1593	-0.00323	0.51342
C	-3.89917	-0.61748	0.16787
N	-2.92809	0.34337	0.01706
H	-5.65366	2.11724	0.77071
H	-6.07343	-0.53897	0.72164
C	-3.71895	-1.98184	-0.03088
C	-4.91281	-2.87238	-0.00116
C	-4.99255	-3.92481	0.91918
C	-5.97374	-2.67162	-0.89239
C	-6.10875	-4.75556	0.94882
H	-4.17545	-4.08268	1.61701
C	-7.08867	-3.50472	-0.86443
H	-5.91425	-1.86253	-1.61444
C	-7.1589	-4.54804	0.05638
H	-6.15951	-5.56309	1.6718
H	-7.90045	-3.34082	-1.5655
H	-8.02827	-5.19693	0.07848
C	3.91051	-2.05477	1.20735
H	3.52762	-2.95089	1.68823
C	5.22612	-1.8073	1.26964
H	5.85289	-2.47017	1.86132
C	5.91553	-0.69023	0.58753
C	6.84123	0.0978	1.28254
H	7.07853	-0.12614	2.31997
C	6.2622	0.58688	-1.25894
N	5.6499	-0.4565	-0.70514
N	7.45175	1.14448	0.72851
C	7.14533	1.39646	-0.54483
H	7.6162	2.25473	-1.01523
H	6.05288	0.78965	-2.3059
H	0.55301	-0.18011	-1.31682
Ni	-1.00369	0.04251	-0.0598

Ni-NCTPP-p_b.trans

C	-0.50623	2.54797	0.05899
C	-0.36206	3.98361	0.08665
C	0.96074	4.24938	0.0219
C	1.63759	2.9721	-0.00852
C	2.8099	-2.5376	0.03672
C	2.70877	-3.97762	0.03939
C	1.38816	-4.27255	0.02023
C	0.68161	-3.01898	-0.01269
C	-0.69943	-2.92058	-0.02827
C	-1.3089	-1.6539	-0.00624
C	-3.04699	-0.24296	-0.1031
C	-0.71712	-0.38949	0.05734
C	-1.82574	0.50257	0.01248
C	-1.73345	1.91681	0.07148
C	-1.54771	-4.14178	-0.06842
C	-2.96609	2.74605	0.17971
C	-3.39033	3.55337	-0.88035
C	-4.57464	4.2807	-0.78007
C	-5.33824	4.21676	0.38359
C	-4.9144	3.42368	1.44895
C	-3.73723	2.68886	1.34608
H	-1.18103	4.68564	0.13994
H	1.44974	5.21141	0.00308
H	3.54585	-4.65881	0.05586
H	0.92199	-5.24682	0.03269
H	-2.79553	3.59485	-1.78812
H	-4.9007	4.89562	-1.61255
H	-6.26141	4.78193	0.45975
H	-5.5037	3.37342	2.35873
H	-3.41081	2.05837	2.16821
N	0.75093	1.94124	0.00815
N	1.57782	-1.95539	0.00496
N	-2.69771	-1.53197	-0.0983
H	-3.33172	-2.30386	-0.26198
C	-1.4498	-5.05388	-1.12674
H	-0.73627	-4.86708	-1.92357
C	-2.4852	-4.37883	0.94648
C	-2.26523	-6.18105	-1.16394
H	-2.18082	-6.87794	-1.99126
C	-3.19169	-6.41029	-0.14831
H	-3.82738	-7.2889	-0.17993
C	-3.30005	-5.50703	0.90671
H	-4.01511	-5.68203	1.70372
H	-2.56024	-3.68213	1.77695
C	4.04921	-1.8668	0.04184
C	4.15591	-0.48843	0.00483
C	5.29813	-2.68431	0.08108
C	6.17017	-2.70703	-1.01281
C	5.6193	-3.43959	1.2148
C	7.33855	-3.4642	-0.97359
H	5.92482	-2.12593	-1.89728
C	6.78483	-4.19999	1.25374
H	4.94782	-3.42506	2.06872
C	7.64803	-4.2134	0.15948
H	8.00436	-3.47187	-1.83058
H	7.02145	-4.7782	2.14112
H	8.55721	-4.80493	0.19012
C	5.42998	0.21297	0.03554
C	5.1505	1.52871	-0.00026
C	3.70136	1.64834	-0.04569
N	3.11467	0.40636	-0.03429
H	6.39632	-0.26559	0.09347
H	5.83765	2.36147	0.02218
C	3.04311	2.86419	-0.04597
C	3.85474	4.11741	-0.07075
C	3.88226	4.97475	1.03533
C	4.61099	4.44997	-1.19981
C	4.64589	6.1386	1.0117
H	3.3011	4.72075	1.9174
C	5.37861	5.61194	-1.22322
H	4.59286	3.78931	-2.06197
C	5.39667	6.45975	-0.11788
H	4.65892	6.79188	1.87821
H	5.95922	5.85634	-2.10691
H	5.99327	7.36596	-0.13582
C	-4.43877	0.14986	-0.25581
H	-4.65103	1.11133	-0.70966
C	-5.46558	-0.62414	0.13469
H	-5.29337	-1.56566	0.65234
C	-6.86855	-0.24647	-0.05686
C	-7.89364	-1.06948	0.42983
H	-7.65278	-1.99523	0.94706
C	-8.44005	1.19894	-0.83867
N	-7.15466	0.89669	-0.69922
N	-9.18273	-0.76742	0.28698
C	-9.45445	0.37246	-0.34897
H	-10.49877	0.64079	-0.47658
H	-8.68232	2.12374	-1.35485
Ni	1.17755	0.00123	0.01528

Ni-NCTPP-p_b.cis

C	-0.33229	-2.94417	0.25547
C	-0.96998	-4.23915	0.29285
C	-2.28729	-4.03854	0.06915
C	-2.47294	-2.61031	-0.04864
C	-1.68852	2.95018	0.20657
C	-1.10204	4.26455	0.31522
C	0.24003	4.09065	0.31345
C	0.47793	2.67559	0.19082
C	1.74137	2.10864	0.14956
C	1.88021	0.70933	0.11284
C	3.03299	-1.19692	0.02171
C	0.88813	-0.27639	0.20733
C	1.62541	-1.49061	0.19858
C	1.03797	-2.7779	0.32555
C	2.94537	2.98289	0.1999
C	1.87779	-3.97894	0.59441
C	2.02848	-4.99238	-0.35762
C	2.86039	-6.08005	-0.10192
C	3.54127	-6.16994	1.11069
C	3.38441	-5.17051	2.06998
C	2.55999	-4.07929	1.81242
H	-0.45916	-5.17744	0.44926
H	-3.07314	-4.77509	-0.00089
H	-1.65676	5.1883	0.37975
H	1.00876	4.84504	0.39229
H	1.50595	-4.91228	-1.3063
H	2.97961	-6.85468	-0.85236
H	4.1909	-7.01626	1.308
H	3.90802	-5.23875	3.01793
H	2.44756	-3.29033	2.55042
N	-1.29098	-1.9475	0.06762
N	-0.72871	1.98507	0.14271
N	3.13704	0.13132	-0.00562
H	4.04317	0.60113	-0.19682
C	3.17074	3.95944	-0.77841
H	2.48158	4.04232	-1.61336
C	3.85376	2.8656	1.26106
C	4.25644	4.82567	-0.67658
H	4.41445	5.58154	-1.43875
C	5.13263	4.72618	0.40328
H	5.9722	5.40825	0.4881
C	4.93425	3.73748	1.36618
H	5.61792	3.64768	2.20393
H	3.68976	2.10716	2.02129
C	-3.08098	2.74921	0.11895
C	-3.64728	1.49901	-0.04806
C	-3.9737	3.9448	0.17948
C	-4.69466	4.35566	-0.94689
C	-4.10233	4.67595	1.36589
C	-5.53039	5.46839	-0.88771
H	-4.59479	3.79574	-1.87248
C	-4.93387	5.79117	1.42506
H	-3.54551	4.3624	2.24448
C	-5.65117	6.18932	0.29834
H	-6.08295	5.77451	-1.77018
H	-5.02526	6.34632	2.35311
H	-6.30051	7.05748	0.3446
C	-5.0797	1.27967	-0.17754
C	-5.25893	-0.04705	-0.31493
C	-3.93991	-0.65723	-0.24697
N	-2.97205	0.30372	-0.08968
H	-5.82785	2.05801	-0.15153
H	-6.18503	-0.59203	-0.42344
C	-3.74035	-2.02543	-0.24715
C	-4.92375	-2.91824	-0.42881
C	-5.4036	-3.70192	0.62687
C	-5.58422	-2.97017	-1.66094
C	-6.51471	-4.52269	0.45251
H	-4.90039	-3.66112	1.58889
C	-6.6986	-3.78746	-1.83538
H	-5.21725	-2.363	-2.48362
C	-7.16528	-4.56711	-0.77935
H	-6.87637	-5.12287	1.28124
H	-7.19967	-3.81717	-2.7976
H	-8.03242	-5.20523	-0.91473
C	4.1609	-2.08157	-0.20536
H	3.91198	-3.13213	-0.15336
C	5.46197	-1.8448	-0.50204
H	6.05963	-2.74427	-0.62627
C	6.24536	-0.63234	-0.73401
C	7.60317	-0.77792	-1.07851
H	8.04331	-1.77018	-1.13661
C	6.49915	1.63795	-0.93666
N	5.71643	0.59849	-0.65719
N	8.39434	0.25377	-1.34525
C	7.83735	1.46659	-1.28069
H	8.46705	2.32193	-1.50344
H	6.0489	2.62433	-0.88101
Ni	-1.02186	0.01383	0.09613

Zn-TPP

N	0.00021	2.05152	0.00037
N	2.05163	-0.00021	0.0421
N	-0.00022	-2.05157	0.00049
N	-2.05164	0.00019	-0.0421
C	-1.09926	2.86494	0.00701
C	-0.67814	4.24923	-0.00234
C	0.67895	4.24911	0.00295
C	1.09981	2.86475	-0.00635
C	2.43979	2.43944	-0.01206
C	2.86375	1.099	-0.00706
C	4.24492	0.6781	-0.10173
C	4.24479	-0.67897	-0.10153
C	2.86353	-1.09958	-0.00683
C	2.43931	-2.43994	-0.01168
C	1.09924	-2.86499	-0.00615
C	0.67811	-4.24928	0.00297
C	-0.67898	-4.24914	-0.00263
C	-1.09983	-2.86477	0.00681
C	-2.43982	-2.43947	0.01178
C	-2.86378	-1.09903	0.00654
C	-4.24503	-0.67814	0.10038
C	-4.24489	0.67893	0.1005
C	-2.86357	1.09955	0.00671
C	-2.43933	2.4399	0.0121
H	-1.33855	5.10366	-0.01181
H	1.33951	5.10342	0.01226
H	5.09537	1.33937	-0.17882
H	5.09511	-1.34043	-0.17844
H	1.3385	-5.10372	0.01236
H	-1.33955	-5.10344	-0.01233
H	-5.09552	-1.33942	0.17677
H	-5.09526	1.34036	0.17699
Zn	0.00002	-0.00003	0.00231
C	-3.49783	3.49345	0.03632
C	-4.38636	3.63307	-1.036
C	-3.62242	4.35505	1.1322
C	-5.37572	4.6125	-1.01387
H	-4.29359	2.96924	-1.89077
C	-4.61115	5.33503	1.15455
H	-2.94007	4.24821	1.97057
C	-5.4902	5.46629	0.08134
H	-6.0547	4.71081	-1.85471
H	-4.6974	5.99312	2.01311
H	-6.26103	6.22978	0.0987
C	-3.49855	-3.4928	0.03561
C	-3.62347	-4.3547	1.13122
C	-4.38701	-3.63185	-1.03683
C	-4.61247	-5.3344	1.15318
H	-2.94117	-4.24829	1.96968
C	-5.37665	-4.61101	-1.01509
H	-4.29399	-2.96779	-1.89139
C	-5.49146	-5.4651	0.07985
H	-4.69899	-5.99272	2.01154
H	-6.05558	-4.70888	-1.85602
H	-6.26252	-6.22838	0.09692
C	3.49786	-3.49345	-0.03597
C	4.38678	-3.63266	1.03608
C	3.62218	-4.35534	-1.13165
C	5.37623	-4.612	1.01389
H	4.29425	-2.96858	1.89068
C	4.611	-5.33522	-1.15406
H	2.93954	-4.24879	-1.96983
C	5.49042	-5.46609	-0.08111
H	6.05551	-4.70999	1.85452
H	4.69702	-5.99354	-2.01247
H	6.26133	-6.22951	-0.09852
C	3.4985	3.49277	-0.03669
C	3.62271	4.35447	-1.13254
C	4.38766	3.63204	1.03516
C	4.61168	5.33418	-1.15534
H	2.93986	4.24788	-1.97054
C	5.37727	4.61121	1.01258
H	4.29521	2.96813	1.88989
C	5.49136	5.46508	-0.0826
H	4.69763	5.99233	-2.01389
H	6.05675	4.70924	1.85304
H	6.2624	6.22837	-0.10031

Zn-NCTPP_a

C	1.31407	2.84772	-0.00926
C	0.97484	4.25991	0.00151
C	-0.37871	4.34706	0.02257
C	-0.88281	2.983	0.00432
C	-1.31868	-2.83364	-0.03283
C	-1.01266	-4.25349	-0.02172
C	0.34184	-4.3572	-0.02781
C	0.8719	-3.00759	-0.02659
C	2.2272	-2.66647	-0.0363
C	2.67911	-1.3184	-0.10604
C	4.16207	0.34207	0.13819
H	5.11837	0.80802	0.33337
C	1.95069	-0.14265	-0.26523
C	2.88338	0.91549	-0.09839
C	2.60364	2.32254	-0.0338
C	3.24733	-3.74438	0.0667
C	3.76196	3.25329	0.03588
C	3.89187	4.17606	1.08243
C	4.99139	5.02715	1.14439
C	5.97886	4.96906	0.16278
C	5.86338	4.05179	-0.87986
C	4.76666	3.19725	-0.9403
H	1.68364	5.07533	-0.01611
H	-0.98232	5.24253	0.04475
H	-1.73602	-5.05563	-0.00957
H	0.93066	-5.26332	-0.04285
H	3.13079	4.21011	1.85605
H	5.08001	5.73151	1.96511
H	6.8355	5.6332	0.21187
H	6.62672	4.00254	-1.64952
H	4.67324	2.48779	-1.75734
N	0.13892	2.11049	-0.00812
N	-0.18086	-2.11387	-0.03073
N	4.03193	-0.98058	0.12554
H	4.78328	-1.63706	0.29584
C	3.22984	-4.65392	1.13255
H	2.45653	-4.56739	1.88961
C	4.2664	-3.84609	-0.89131
C	4.19974	-5.64673	1.23088
H	4.17521	-6.3411	2.0643
C	5.20403	-5.74347	0.26939
H	5.96035	-6.51737	0.34812
C	5.23529	-4.84084	-0.79153
H	6.01153	-4.91284	-1.54619
H	4.28305	-3.15188	-1.72707
C	-2.62752	-2.27019	-0.02742
C	-2.91428	-0.90399	-0.02273
C	-3.77518	-3.22649	-0.02578
C	-4.65864	-3.28277	1.05869
C	-3.98597	-4.09105	-1.10697
C	-5.72886	-4.17391	1.06022
H	-4.49853	-2.62074	1.90503
C	-5.05248	-4.98617	-1.10498
H	-3.30749	-4.0534	-1.95467
C	-5.92807	-5.02912	-0.02149
H	-6.40365	-4.20359	1.90974
H	-5.20315	-5.6461	-1.95335
H	-6.76047	-5.72527	-0.02047
C	-4.26975	-0.36538	-0.07155
C	-4.16938	0.97931	-0.06154
C	-2.74875	1.30889	-0.00687
N	-2.0194	0.141	0.00227
H	-5.17063	-0.95882	-0.12403
H	-4.97174	1.70107	-0.1039
C	-2.26184	2.61613	0.00628
C	-3.25474	3.73265	0.01509
C	-3.33703	4.61963	-1.06532
C	-4.11526	3.92067	1.10297
C	-4.2545	5.66673	-1.05859
H	-2.67542	4.48022	-1.91576
C	-5.03718	4.96469	1.10933
H	-4.05438	3.24096	1.94825
C	-5.10835	5.84154	0.02899
H	-4.30632	6.3429	-1.90597
H	-5.69596	5.09605	1.96179
H	-5.8246	6.65669	0.03445
Zn	-0.03005	0.00315	-0.2766

Zn-NCTPP_b

C	-1.07129	2.89964	0.2471
C	-0.59883	4.25771	0.32499
C	0.75594	4.2163	0.24031
C	1.12971	2.82693	0.12235
C	1.03863	-2.84779	0.14408
C	0.61715	-4.22271	0.28127
C	-0.73882	-4.21839	0.33901
C	-1.16682	-2.846	0.22782
C	-2.50905	-2.43877	0.16846
C	-2.87644	-1.07558	0.18514
C	-4.03284	0.66092	-0.38205
H	-4.8094	1.26106	-0.84544
C	-2.10702	0.02708	0.61411
C	-2.83109	1.17908	0.23492
C	-2.42475	2.52792	0.21807
C	-3.57787	-3.45106	-0.01605
C	-3.47571	3.56388	0.05814
C	-3.38411	4.56181	-0.92306
C	-4.39725	5.50319	-1.07255
C	-5.51993	5.46223	-0.24721
C	-5.62869	4.46831	0.72331
C	-4.61756	3.52386	0.87108
H	-1.22559	5.12862	0.44837
H	1.4426	5.04933	0.24818
H	1.27606	-5.07721	0.31658
H	-1.39594	-5.06586	0.46642
H	-1.38355	-0.00988	1.42381
H	-2.52241	4.58087	-1.583
H	-4.31454	6.26401	-1.84181
H	-6.30891	6.19786	-0.36445
H	-6.50046	4.42921	1.3682
H	-4.69741	2.75232	1.63107
N	0.0093	2.04214	0.14389
N	-0.05611	-2.02772	0.13038
N	-4.06276	-0.6482	-0.41784
C	-3.48112	-4.45082	-0.99317
H	-2.61019	-4.47841	-1.64082
C	-4.72835	-3.40228	0.78338
C	-4.49831	-5.38753	-1.15351
H	-4.41035	-6.15092	-1.91974
C	-5.62828	-5.33926	-0.34018
H	-6.42011	-6.07094	-0.46362
C	-5.74125	-4.34185	0.62787
H	-6.62024	-4.29677	1.2627
H	-4.81486	-2.62317	1.53421
C	2.39382	-2.45993	0.01915
C	2.85014	-1.14272	-0.09436
C	3.42982	-3.53623	0.04313
C	4.22823	-3.77489	-1.08102
C	3.63484	-4.30978	1.19165
C	5.20645	-4.76598	-1.05951
H	4.07487	-3.17782	-1.97549
C	4.61056	-5.30244	1.21322
H	3.02478	-4.12452	2.07121
C	5.39894	-5.53322	0.08725
H	5.81554	-4.94058	-1.94058
H	4.75906	-5.8919	2.11223
H	6.16025	-6.30623	0.10439
C	4.24418	-0.73987	-0.07099
C	4.2663	0.61486	-0.08094
C	2.88607	1.06247	-0.10875
N	2.06155	-0.0279	-0.13597
H	5.08549	-1.4153	-0.0183
H	5.12923	1.2631	-0.03718
C	2.47202	2.39346	-0.00958
C	3.54198	3.43619	-0.01009
C	3.78012	4.22225	1.12356
C	4.34141	3.62752	-1.14248
C	4.79073	5.17958	1.12287
H	3.16918	4.07373	2.00944
C	5.35408	4.58361	-1.14336
H	4.16214	3.02063	-2.02542
C	5.58043	5.36275	-0.01091
H	4.96534	5.77863	2.01078
H	5.96366	4.72153	-2.03055
H	6.36902	6.1081	-0.01086
Zn	0.06253	0.00619	-0.19961

Zn-NCTPP-p_a.trans

C	-0.6198	2.52278	-0.76218
C	-0.46744	3.93747	-0.99273
C	0.82842	4.24887	-0.73057
C	1.48473	3.02344	-0.33843
C	2.82244	-2.47665	-0.06596
C	2.79522	-3.9162	-0.23577
C	1.5114	-4.26084	-0.49533
C	0.73459	-3.04016	-0.51311
C	-0.65503	-2.99467	-0.62834
C	-1.36767	-1.76566	-0.61426
C	-2.99147	-0.38995	-0.18324
C	-0.86946	-0.49617	-0.97922
C	-1.90778	0.42402	-0.7144
C	-1.83858	1.8317	-0.83588
C	-1.41638	-4.27435	-0.705
C	-3.09954	2.59426	-1.00453
C	-4.02426	2.18644	-1.97634
C	-5.24544	2.83818	-2.10921
C	-5.56526	3.90115	-1.26572
C	-4.65465	4.31386	-0.29453
C	-3.42795	3.6687	-0.16655
H	-1.2483	4.59903	-1.33816
H	1.30429	5.21527	-0.80527
H	3.64999	-4.56994	-0.14653
H	1.11458	-5.25141	-0.66168
H	-3.77597	1.35033	-2.62331
H	-5.94968	2.51421	-2.86841
H	-6.52252	4.403	-1.36168
H	-4.9034	5.13319	0.37189
H	-2.73017	3.97157	0.6081
N	0.60333	1.98632	-0.39727
N	1.57501	-1.9652	-0.26367
N	-2.65824	-1.67405	-0.145
C	-1.25727	-5.11264	-1.81449
H	-0.58203	-4.81536	-2.61181
C	-2.29816	-4.65808	0.31224
C	-1.95717	-6.31362	-1.9033
H	-1.82258	-6.95165	-2.77079
C	-2.82802	-6.6904	-0.88413
H	-3.3739	-7.62586	-0.95113
C	-2.9957	-5.85916	0.22236
H	-3.67101	-6.14815	1.02141
H	-2.43337	-4.00684	1.16821
C	3.99528	-1.75777	0.27159
C	4.08219	-0.36493	0.37241
C	5.23742	-2.54664	0.52959
C	6.3558	-2.41446	-0.30137
C	5.30623	-3.42889	1.61462
C	7.51579	-3.14384	-0.05233
H	6.30861	-1.73654	-1.14888
C	6.46397	-4.16104	1.8626
H	4.44363	-3.53459	2.26652
C	7.57242	-4.01962	1.02983
H	8.37349	-3.0313	-0.7076
H	6.50171	-4.83827	2.70977
H	8.4755	-4.5892	1.22337
C	5.28182	0.38109	0.71576
C	4.96419	1.69582	0.65205
C	3.56501	1.77896	0.26769
N	3.06213	0.51167	0.14044
H	6.23501	-0.05636	0.9739
H	5.60761	2.54065	0.84929
C	2.85581	2.95635	0.03196
C	3.59297	4.24859	0.15473
C	3.19977	5.20375	1.10037
C	4.68791	4.53038	-0.67066
C	3.88676	6.4083	1.22068
H	2.35161	4.99245	1.74538
C	5.37665	5.7347	-0.54997
H	4.99263	3.79949	-1.41419
C	4.97805	6.67649	0.39613
H	3.5722	7.13577	1.96199
H	6.22147	5.93891	-1.19978
H	5.51389	7.61539	0.48986
C	-4.26548	0.06958	0.36047
H	-4.41058	1.13396	0.50822
C	-5.27042	-0.76269	0.67709
H	-5.15178	-1.83358	0.53256
C	-6.55526	-0.29748	1.20231
C	-7.56388	-1.2194	1.52331
H	-7.38976	-2.28434	1.38782
C	-7.94907	1.38715	1.83718
N	-6.76246	1.02008	1.36566
N	-8.75221	-0.85069	1.99592
C	-8.94414	0.46043	2.15251
H	-9.90663	0.78647	2.53498
H	-8.12472	2.45136	1.96997
H	-0.12492	-0.35046	-1.75851
Zn	1.12082	0.0301	0.02841

Zn-NCTPP-p_a.cis

C	-0.31686	-2.9772	-0.36919
C	-0.98751	-4.2282	-0.61186
C	-2.32193	-3.99542	-0.49988
C	-2.47933	-2.59543	-0.1864
C	-1.57961	3.00502	-0.10098
C	-0.97249	4.30997	-0.27895
C	0.36395	4.11501	-0.38264
C	0.59582	2.69018	-0.29711
C	1.85649	2.09692	-0.25962
C	2.01295	0.68917	-0.17444
C	2.93675	-1.18593	0.41181
C	1.10261	-0.30127	-0.59582
C	1.6746	-1.54021	-0.22103
C	1.07822	-2.81527	-0.31865
C	3.07778	2.95394	-0.26025
C	1.95388	-4.01115	-0.31865
C	3.07593	-4.04189	-1.15919
C	3.94078	-5.13064	-1.14654
C	3.70545	-6.19963	-0.28354
C	2.59841	-6.17668	0.5635
C	1.72639	-5.09255	0.54495
H	-0.49885	-5.15694	-0.86773
H	-3.12903	-4.70097	-0.62933
H	-1.50792	5.24747	-0.30156
H	1.13419	4.86156	-0.51024
H	3.26314	-3.2015	-1.82124
H	4.80128	-5.14321	-1.80733
H	4.3846	-7.04573	-0.26729
H	2.41817	-7.00025	1.24653
H	0.87845	-5.0629	1.2222
N	-1.259	-1.98958	-0.14335
N	-0.61826	2.03921	-0.13734
N	3.11292	0.12392	0.43436
C	3.39007	3.71939	-1.38964
H	2.72975	3.68793	-2.25168
C	3.9424	2.98969	0.84068
C	4.53891	4.50734	-1.41818
H	4.76706	5.09319	-2.3028
C	5.39244	4.53711	-0.31842
H	6.29001	5.14689	-0.34029
C	5.08962	3.77637	0.81022
H	5.75454	3.78804	1.66818
H	3.71465	2.38617	1.71173
C	-2.96888	2.81469	0.08682
C	-3.60069	1.57005	0.19219
C	-3.83282	4.03255	0.14059
C	-4.78286	4.27282	-0.85891
C	-3.71053	4.95348	1.18761
C	-5.5932	5.4042	-0.81216
H	-4.87939	3.56565	-1.67785
C	-4.51821	6.08681	1.23396
H	-2.97764	4.77304	1.96897
C	-5.46221	6.31459	0.23436
H	-6.32292	5.57682	-1.59667
H	-4.41291	6.78983	2.05397
H	-6.09209	7.19741	0.27083
C	-5.02841	1.36749	0.3716
C	-5.24508	0.03075	0.35767
C	-3.95468	-0.60867	0.16656
N	-2.98297	0.35603	0.10914
H	-5.75857	2.15419	0.49254
H	-6.18696	-0.48666	0.46636
C	-3.744	-1.97873	0.01694
C	-4.94058	-2.87169	0.03223
C	-5.0801	-3.85767	1.01653
C	-5.93984	-2.74517	-0.93989
C	-6.19289	-4.69386	1.03067
H	-4.3089	-3.96245	1.77436
C	-7.05381	-3.58071	-0.92602
H	-5.83357	-1.98982	-1.71323
C	-7.18322	-4.55701	0.05946
H	-6.28823	-5.45031	1.80292
H	-7.81747	-3.47212	-1.68938
H	-8.05042	-5.20914	0.06982
C	3.87808	-2.07527	1.10516
H	3.46256	-2.97732	1.54645
C	5.1848	-1.83242	1.27981
H	5.75666	-2.50585	1.9139
C	5.93634	-0.70583	0.68404
C	6.77389	0.08242	1.48385
H	6.88845	-0.14447	2.54132
C	6.4932	0.57939	-1.10479
N	5.82095	-0.46623	-0.62957
N	7.44535	1.13015	1.00803
C	7.28967	1.38515	-0.29193
H	7.81346	2.24353	-0.70251
H	6.40566	0.78618	-2.16816
H	0.41797	-0.17981	-1.43296
Zn	-1.00556	0.03738	0.2294

Zn-NCTPP-p_b.trans

C	-0.53921	2.6245	0.14044
C	-0.39938	4.07167	0.14922
C	0.92391	4.34734	0.04784
C	1.61221	3.06621	0.00842
C	2.83704	-2.62652	0.00899
C	2.73085	-4.0755	0.01554
C	1.40532	-4.36601	0.06429
C	0.69341	-3.10231	0.0704
C	-0.69321	-2.95174	0.1224
C	-1.32721	-1.67775	0.19632
C	-3.05901	-0.2505	-0.01205
C	-0.77426	-0.40733	0.3456
C	-1.85348	0.50358	0.20918
C	-1.74736	1.93836	0.2146
C	-1.56058	-4.15976	0.07616
C	-2.996	2.74199	0.32477
C	-3.39807	3.60968	-0.69699
C	-4.59618	4.31329	-0.59405
C	-5.39872	4.16705	0.53546
C	-5.00077	3.31213	1.56251
C	-3.81094	2.59961	1.45497
H	-1.21248	4.77927	0.22409
H	1.39576	5.31824	0.00989
H	3.55856	-4.76895	-0.01296
H	0.94847	-5.34457	0.1034
H	-2.77604	3.71257	-1.58124
H	-4.90337	4.97309	-1.3989
H	-6.33246	4.71428	0.61384
H	-5.62069	3.19621	2.44557
H	-3.50531	1.92148	2.24659
N	0.7245	2.05869	0.05165
N	1.6111	-2.07065	0.03535
N	-2.71005	-1.54612	0.00189
H	-3.33602	-2.30589	-0.23411
C	-1.4604	-5.08626	-0.97027
H	-0.73443	-4.91742	-1.75965
C	-2.52196	-4.37139	1.07552
C	-2.29179	-6.20172	-1.00871
H	-2.20504	-6.90817	-1.82766
C	-3.23812	-6.40622	-0.00629
H	-3.88613	-7.27579	-0.03872
C	-3.35173	-5.48805	1.03563
H	-4.08334	-5.64268	1.8218
H	-2.60278	-3.66246	1.89487
C	4.05599	-1.89078	-0.03686
C	4.1528	-0.49912	-0.05171
C	5.32222	-2.68336	-0.06991
C	6.16334	-2.64724	-1.18817
C	5.68729	-3.48544	1.01809
C	7.34228	-3.38821	-1.21622
H	5.88434	-2.03273	-2.0395
C	6.86309	-4.23041	0.98993
H	5.04194	-3.51803	1.89149
C	7.69481	-4.1828	-0.12743
H	7.98237	-3.34871	-2.09179
H	7.13251	-4.84391	1.8437
H	8.61206	-4.76216	-0.14939
C	5.42365	0.21866	-0.06573
C	5.14034	1.53661	-0.07687
C	3.6865	1.66827	-0.06649
N	3.1226	0.41316	-0.03723
H	6.39817	-0.24693	-0.05416
H	5.83696	2.362	-0.07607
C	3.02624	2.8966	-0.05965
C	3.85581	4.13801	-0.11745
C	3.8792	5.03213	0.95982
C	4.62193	4.43415	-1.25087
C	4.64802	6.19164	0.90553
H	3.29072	4.80932	1.84552
C	5.39555	5.59099	-1.305
H	4.60539	3.74846	-2.09335
C	5.40952	6.47373	-0.22718
H	4.65689	6.87204	1.75105
H	5.98324	5.80489	-2.19199
H	6.01019	7.37644	-0.26935
C	-4.43455	0.13928	-0.2783
H	-4.60814	1.11416	-0.72035
C	-5.49378	-0.64101	-0.00359
H	-5.37163	-1.59878	0.49842
C	-6.87266	-0.24794	-0.3066
C	-7.94053	-1.08125	0.05522
H	-7.75008	-2.02679	0.55743
C	-8.36388	1.23533	-1.17188
N	-7.09703	0.92006	-0.9289
N	-9.21051	-0.76598	-0.19133
C	-9.42076	0.39828	-0.80644
H	-10.4486	0.67771	-1.01703
H	-8.55571	2.18018	-1.67272
Zn	1.17832	-0.00078	0.32428

Zn-NCTPP-p_b.cis

C	-0.3421	-3.039	0.13448
C	-0.98568	-4.34319	0.16576
C	-2.31836	-4.13738	0.0258
C	-2.50835	-2.69769	-0.0526
C	-1.66044	3.06365	0.1075
C	-1.05907	4.3816	0.21845
C	0.28117	4.18845	0.31513
C	0.51229	2.75778	0.23623
C	1.75727	2.12804	0.27893
C	1.90652	0.70949	0.24969
C	3.03565	-1.20489	-0.04433
C	0.94192	-0.2945	0.36573
C	1.63964	-1.51535	0.20456
C	1.0306	-2.82078	0.2253
C	2.98258	2.96647	0.39373
C	1.89618	-4.01656	0.42484
C	1.98774	-5.02868	-0.53723
C	2.84261	-6.11122	-0.3427
C	3.60638	-6.20014	0.81943
C	3.51145	-5.20259	1.78903
C	2.66639	-4.11573	1.59084
H	-0.47948	-5.2908	0.27897
H	-3.10138	-4.88053	-0.01094
H	-1.59265	5.32072	0.22546
H	1.04381	4.94408	0.436
H	1.40328	-4.94865	-1.44898
H	2.91571	-6.88235	-1.10264
H	4.27323	-7.04288	0.96897
H	4.10011	-5.26915	2.69818
H	2.60186	-3.32847	2.33633
N	-1.32304	-2.06817	0.0011
N	-0.7058	2.11524	0.11606
N	3.15075	0.12787	0.0166
H	4.0513	0.5975	-0.19358
C	3.26254	3.99417	-0.51661
H	2.60126	4.14659	-1.36412
C	3.86692	2.7539	1.46191
C	4.37011	4.81921	-0.33515
H	4.56911	5.61493	-1.04548
C	5.21787	4.62562	0.75463
H	6.07485	5.27503	0.90034
C	4.96981	3.58245	1.64603
H	5.63175	3.41735	2.4898
H	3.66394	1.95357	2.16772
C	-3.05636	2.80332	-0.00806
C	-3.6313	1.53516	-0.0882
C	-3.96555	3.98887	-0.04186
C	-4.70853	4.28885	-1.18952
C	-4.08639	4.82689	1.0731
C	-5.55558	5.39407	-1.22056
H	-4.61618	3.64712	-2.06123
C	-4.92847	5.93539	1.04203
H	-3.51595	4.60045	1.96958
C	-5.66651	6.22136	-0.10509
H	-6.12435	5.61163	-2.11894
H	-5.01252	6.57254	1.9166
H	-6.32426	7.0841	-0.12919
C	-5.07157	1.30931	-0.16503
C	-5.26704	-0.02361	-0.21077
C	-3.95274	-0.65717	-0.16077
N	-2.98634	0.31971	-0.07816
H	-5.82082	2.08725	-0.16629
H	-6.20752	-0.55272	-0.25758
C	-3.76817	-2.03914	-0.15954
C	-4.9806	-2.90731	-0.2599
C	-5.36946	-3.71821	0.81304
C	-5.74838	-2.92955	-1.42987
C	-6.49575	-4.53136	0.71844
H	-4.78207	-3.70443	1.72694
C	-6.87828	-3.73845	-1.52451
H	-5.45112	-2.30619	-2.26849
C	-7.25402	-4.5429	-0.45086
H	-6.78456	-5.15155	1.56097
H	-7.46198	-3.74352	-2.43948
H	-8.1329	-5.17508	-0.52439
C	4.13583	-2.07249	-0.42379
H	3.8621	-3.1175	-0.47905
C	5.42973	-1.83251	-0.74774
H	5.99978	-2.72443	-0.99539
C	6.23546	-0.61859	-0.86567
C	7.59111	-0.7553	-1.22315
H	8.01262	-1.74557	-1.37618
C	6.53139	1.65549	-0.85202
N	5.72809	0.60893	-0.67513
N	8.4019	0.28202	-1.38927
C	7.86725	1.49345	-1.2083
H	8.51326	2.35432	-1.34734
H	6.09869	2.64022	-0.70431
Zn	-1.02581	0.01084	0.30249
